# ddRAD-seq derived genome-wide SNPs, high density linkage map and QTLs for fruit quality traits in strawberry (*Fragaria* x *ananassa*)

**DOI:** 10.1007/s13205-020-02291-5

**Published:** 2020-07-27

**Authors:** Sathishkumar Natarajan, Mohammad Rashed Hossain, Hoy-Taek Kim, Michael Immanuel Jesse Denison, Mostari Jahan Ferdous, Hee-Jeong Jung, Jong-In Park, Ill-Sup Nou

**Affiliations:** Department of Horticulture, Suncheon National University, 255 Jungang-ro, Suncheon, Jeonnam 57922 Republic of Korea

**Keywords:** Strawberry, Fruit quality, Sugar content, ddRAD-seq, Linkage map, QTL, SNP, Marker

## Abstract

Understanding the genetic determinants are essential for improving the fruit quality traits of strawberry. In this study, we focused on mapping the loci for fruit-length (FL), -diameter (FD), -weight (FW) and -soluble solid content (SSC) using the genome-wide single nucleotide polymorphisms (SNPs) identified via ddRAD-sequencing of the F_1_ population raised from Maehyang (♀) X Festival (♂). A total of 12,698 high quality SNPs were identified of which 1554 SNPs that showed significant Mendelian segregation (*p* < 0.05) were mapped to 53 linkage groups (LG) spanning a total of 2937.93 cM with an average marker density of 2.14 cM/locus. Six QTLs for FL and four QTLs for each of FD, FW and SSC were identified that explained 24–35%, 21–42%, 24–54% and 23–50% of overall phenotypic variations, respectively. The genes that lie within these QTL regions were extracted and discussed thoroughly. In addition, a high resolution melting marker (MF154) were designed based on the SNP A1^723^G of the UDP-glucose 4-epimerase GEPI48-like gene FAN_iscf00021287. The marker detected the high vs low sugar containing F_1_ plants and commercial cultivars with 81.39% and 86.95% detection accuracy, respectively. These SNPs, linkage map, QTLs and candidate genes will be helpful in understanding and improving the fruit quality traits of strawberry.

**Electronic supplementary material:**

The online version of this article (10.1007/s13205-020-02291-5) contains supplementary material, which is available to authorized users.

## Introduction

The cultivated strawberry (*Fragaria* × *ananassa*), an economically important berry crop, is favored across the globe due to its characteristic aesthetic property, flavor, nutritional value and health benefitting anti-oxidative content (Alvarez-Suarez et al. 2014; Henning et al. 2010). The increasing popularity of the fruit is evident from 21.21% and 57.1% increase in global cultivation area and production, respectively, during the last decade (FAOSTAT 2017). The increasing demand for the fruit necessitates the improvement of both agronomic and fruit quality attributes of the crop. Wider understanding of the genetic determinants of these traits are thus very important. However, compared to other economically important fruits, genetic control of key traits of cultivated strawberry is yet to be completely understood.

*Fragaria* × *ananassa* has undergone complex evolutionary changes during its evolutionary journey starting from wild diploid progenitors to the current cultivated octoploid (2*n* = 8 ×  = 56) species (Potter et al. 2000). Evidences from recent studies indicate three diploid progenitors namely, *F. vesca, F. nubicola* and *F. orientalis;* or *F. orientalis, F. iinumae* and *F. vesca or F. mandshurica* (Edger et al. 2019; Rousseau-Gueutin and Gaston 2009), which ultimately lead to the extant octoploid species with four relatively similar sub-genomic components (Rousseau-Gueutin and Gaston 2009; Sargent et al. 2016; Tennessen et al. 2014). The genome composition of the current *Fragaria* × *ananassa* is proposed to be either AABBBBCC, AAA′A′BBBB, or AAA′A′BBB′B′, with the latter being supported by most of the studies (Isobe et al. 2013; Kunihisa 2011; Sargent et al. 2012; Shaw 1997). The complexity of the genetics of this species thus can be attributed to the various combinations of gene interactions between the multiple alleles, complex meiotic behaviors and highly heterozygous nature of the species (Lerceteau-Köhler et al. 2003; Rousseau-Gueutin et al. 2008; Spigler et al. 2010; Weebadde et al. 2008).

The complex octoploid genomic structure made it difficult to disentangle the species genetically (Sánchez-Sevilla et al. 2015). However, the recent release of *Fragaria* × *ananassa* scaffolds in 2014 (Hirakawa et al. 2014) and the release of the complete genome of its diploid progenitor *F. vesca* (a simper model species) in 2011 (Shulaev et al. 2011) have facilitated the genetic mapping of economically important traits of cultivated strawberry. Genetic mapping of various important traits, particularly prior to the release of complete and annotated genome, can facilitate the identification of the causal loci (and specific genes, in some cases). For polyploid species, genetic mapping is also helpful in identifying the homoeoalleles and their contribution in governing the complex trait, for example, the identification of homoeoalleles for drought resistance in durum wheat (Peleg et al. 2009) and for cane yield in sugarcane (Aitken et al. 2006). Several homoeologous linkage groups have also been identified in the octoploid cultivated strawberry as well (Rousseau-Gueutin et al. 2008; Sargent et al. 2009).

Linkage mapping in strawberry have initially been constructed with low density non-transferrable markers such as single dose restriction fragment (SDRF) markers in ‘Capitola’ × ‘CF1116’ population (Lerceteau-Köhler et al. 2003) and AFLP markers in ‘Tribute’ × ‘Honeoye’ population (Weebadde et al. 2008). These were followed by the use of a comprehensive suite of sequence characterized and transferrable markers such as RAPD markers in ‘Ever Berry’ × ‘Toyonoka’ population (Sugimoto et al. 2005), combination of AFLP, RAPD, SSR and gene specific markers in ‘Redgauntlet’ × ‘Hapil’ population (Sargent et al. 2009) and combination of SSR, STS and SCAR markers in the extended ‘Capitola’ × ‘CF1116’ population (Rousseau-Gueutin et al. 2008). Several QTLs for various agronomic, reproductive and fruit quality traits have already been identified using these populations (Lerceteau-Köhler et al. 2012; Zorrilla-Fontanesi et al. 2011; Lerceteau-Kohler et al. 2006; Castro and Lewers 2016; Verma et al. 2017). However, the need for high-density linkage maps and finer mapping of QTLs with markers saturating the whole genome were realized by several investigators (Zorrilla-Fontanesi et al. 2011; Castro et al. 2015; Pott et al. 2000).

The draft scaffold of *Fragaria* × *ananassa* have shown high collinearity with its diploid progenitor, *F. vesca* (Rousseau-Gueutin and Gaston 2009; Sargent et al. 2012; Hirakawa et al. 2014). This offers the opportunity to develop genome-wide molecular markers and to construct linkage maps with higher marker density. Moreover, the advancement in the cost-effective and readily available reduced genome sequencing technologies such as restriction site-associated DNA sequencing (RAD-Seq) and genotyping-by-sequencing (GBS) made the identification of the genome-wide SNPs, for this complex octoploid species, more convenient these days (Elshire et al. 2011; Baird et al. 2008). These genome-wide SNPs are helpful in constructing high-density linkage map, which can eventually lead to the identification of novel QTLs and putative functional candidate genes for both diploid and polyploid species (Bassil et al. 2015; Shirasawa et al. 2017). Although, few reports of SNP based linkage mapping in strawberry are available (Sánchez-Sevilla et al. 2015; Davik et al. 2015; Hossain et al. 2019; Vining et al. 2017), no SNP based QTL is reported yet, particularly for fruit quality traits.

In this study, we thus aimed at identifying genome-wide SNPs via ddRAD-seq technique, constructing high density linkage map and mapping QTLs for few fruit quality traits using the F_1_ population raised by crossing two cultivars having contrasting agronomic and economic traits. The findings will enrich our current genome wide SNP knowledgebase for cultivated strawberry and will add new insight in understanding the genetics of the studied fruit quality traits.

## Materials and methods

### Plant materials, growth conditions and phenotyping

Seedlings of high sugar cultivar, Maehyang (♀) and low sugar cultivar Festival (♂) were collected from Damyang-gun Agricultural Technology Center, Damyang, South Korea. The F_1_ population were raised in the green house facility of Sunchon National University, South Korea. The F_1_ seeds were germinated in growth chambers and then transplanted to large (66 cm × 20 cm × 16 cm) rectangular pots filled with commercial nursery soil mixture (50% cocopit and 50% soil) in green house maintaining 25 ± 2 °C, 16 h day length, 80% relative humidity and 440 μmol m^−2^ s^−1^ light intensity. The length (mm) and diameter (mm) of fruits were recorded and the weights (g) were measured using OHAUS balance (model PAG2102, OHAUS Corporation, NJ, USA). Total soluble solid content (SSC) was measured in a refractometer (model PAL-1, ATAGO CO., LTD, Tokyo, Japan) and expressed in °Bx. The field experiment was performed with five replications. Measurements were taken from five uniformly ripe fruits, each (2nd fruit of the 1st fruit cluster) collected from an individual plant. In addition, 23 commercial culitvars (listed in Table S10) were grown and phenotyped for SSC in the green house facility of Damyang-gun Agricultural Technology Center, Damyang, South Korea. A °Bx value of ‘8’ was considered as the cutoff value for high vs low sugar content. These cultivars were used for validating the markers developed for detecting high vs low fruit sugar containing genotypes.

### Isolation of DNA

DNA was collected from the newly emerged young leaves of the parents and F_1_ plants. Fresh leaves were disrupted in TissueLyser II (Qiagen, CA, USA) and DNA was extracted using the protocol of DNeasy Plant Mini Kit (Qiagen, CA, USA). The DNA quality was evaluated by electrophoresis in agarose gel (1.2%) while the concentration and the purity of extracted DNA samples were estimated using Nanodrop-2000 (Nanodrop Technologies, Wilmington, DE, USA).

### Preparation of ddRAD-seq library and sequencing

The ddRAD-seq libraries for all strawberry genotypes were constructed by digesting the genomic DNA (500 ng) with restriction enzymes, *PstI* and *MspI* (New England Biolabs, MA, USA). Unique barcodes of eight nucleotide base pairs and Illumina adaptors were ligated to the digested DNA to enable tracing the samples of each genotype (Table S1). The adaptor-ligated DNA fragments were amplified by 22 cycles of polymerase chain reaction and the amplicons of 300–900 bp length were separated using BluePippin (Sage Science, MA, USA). The extracted DNA fragments were then sequenced by the Illumina HiSeq 2000 platform (Illumina, Inc., CA, USA) using 93 base pair (bp) paired-end (PE) mode following the procedures described in Shirasawa et al. (2017).

### Analysis of sequence data and detection of SNP

Preliminary processing of ddRAD-seq data was performed following the procedures of Shirasawa et al. (2017) with minor modifications. The quality of the data set was controlled using FastQC tool (https://www.bioinformatics.babraham.ac.uk/projects/fastqc/). PRINSEQ (Schmieder and Edwards 2011) and fastx_clipper (FASTX-Toolkit version 0.10.1; https://hannonlab.cshl.edu/fastx_toolkit) were used to filter the low-quality sequences and adaptor sequences, respectively. High quality SNPs were retained based on strict SNP filtration criteria (SNP quality score ≥ 999, minimum depth = 5, minimum allele frequency = 0.05 and maximum proportion of missing data = 0.5) through VCFTool program (https://vcftools.sourceforge.net/).

The high quality reads were then mapped to the *Fragaria* × *ananassa* reference genome sequence (FAN_r1.1, https://strawberry-garden.kazusa.or.jp/) using Bowtie 2 v2.1.0 (parameters: -minins 100 -no-mixed) (Langmead and Salzberg 2012). The resulting SAM (sequence alignment/map) format files were converted into BAM (binary alignment/map) files prior to sorting, indexing and removal of duplicates by SAMtools version 0.1.19 (parameters: -Duf) (Li et al. 2009). The variants from the high-quality alignments were called using mpileup module from SAM tools and BCF tools (parameters: -vcg) view. The resulting variant call format (VCF) files were filtered using VCFtools version 0.1.11 (Danecek et al. 2011) and the missing data were imputed using Beagle4 (Browning and Browning 2007). The SNP associated sequences were retrieved from strawberry reference genome (FAN_r1.1). SnpEff version 4.11 (snpeff.sourceforge.net) was used to predict the effect of SNP annotations on gene functions (Cingolani et al. 2012).

### Construction of linkage map

The SNP markers that were heterozygous either in one parent (segregation genotype code lmxll and nnxnp in JoinMap version 4.1) or in both parents (segregation genotype code hkxhk) were used for linkage mapping. Markers that did not fit significantly with the respective segregation ratio of 1:1 or 1:2:1 in the progeny as per the chi-square test of goodness of fit (*p* < 0.05) were excluded from map construction. Markers showing more than 10% missing data were excluded as well. Marker order and map distances (centi-Morgans) were calculated using regression mapping algorithm and Kosambi’s mapping function (maximum recombination fraction = 0.45, goodness of fit jump threshold = 5 and a ripple value = 1) in JoinMap v4.1 (Ooijen 2006). Based on the minimum logarithm of odds (LOD) score limit of 5.0, linkage groups (LGs) were selected and visualized in MapChart (version 2.32). All LGs were mapped on the *F. vesca* genome v4.0.a1 and the LGs were numbered ‘1’– ‘7’ based on the number of *F. vesca* chromosomes where the LGs were mapped. The suffixes ‘A’- ‘D’ were assigned to the LGs based on their sequential physical position along the length of a particular *F. vesca* chromosome. Two small LGs, if mapped one after another or partially overlapped, to a particular *F. vesca* chromosome, they were considered as one linkage group.

### Identification of QTLs and extracting genes from QTL regions

QTL mapping was performed by integrating the genotypic and phenotypic data of the mapping population through composite interval mapping (CIM) in Windows QTL Cartographer tool version 2.5_011 (parameters: linkage map method = 10, segregation test size = 0.01, linkage test size = 0.35, mapping function = Kosambi, objective function = SAL and step size = 1 cM). Besides, several multi-locus mixed linear models such as mrMLM, pLARmEB and ISIS EM-BLASSO methods were also used to validate the QTLs identified by CIM methods (Tamba et al. 2017; Wang et al. 2016; Wen et al. 2018; Zhang et al. 2017). Putative QTLs were declared based on the significant LOD threshold determined by 1000 permutations. The square of the partial correlation coefficient (*R*^2^) was used to indicate the proportion of phenotypic variation explained by a QTL. Sequence tags associated with the flanking markers of each QTL were mapped to the *Fragaria vesca* annotated genome version v4.0.a1 and the genes within these flanking regions were extracted.

### Development of High resolution melting (HRM) Marker

Five HRM markers were designed to validate their linkage with high vs low sugar contents. These markers were used to genotype the F_1_ population and 23 commercial cultivars in a final reaction volume of 20 μL, containing 50 ng of genomic DNA, 10 μL of ‘HS Prime LP Premix’ (GeNet Bio, Deajeon, Republic of Korea), 0.6 μL of 2xSYTO9 green fluorescent nucleic acid stain (GeNet Bio, Deajeon, Republic of Korea), 0.2, 1.0 and 1.0 μL of forward, reverse and probe primers, respectively (Table 4), and ultra-pure water for the remainder of the volume. High resolution melting was performed in a LightCycler96 (Roche, Mannheim, Germany) using 96-well plate in a 20 μL/well final reaction mix with the cycling condition of initial denaturation at 95 °C for 5 min followed by 40 cycles of three step amplifications at 95 °C for 10 s, 60 °C for 15 s and 72 °C for 15 s. The HRM program included denaturation at 95 °C for 1 min, re-naturation at 40 °C for 2 min and melting from 60 to 90 °C, with a ramp of 0.3 °C per second and five fluorescent acquisitions per degree centigrade. High resolution melting data were analyzed using LightCycler^®^ 96 software v1.1.

### Statistical analysis

Significance test was done using Analysis of variance (ANOVA) and statistically significant differences between cultivars for the fruit quality traits were determined by Students *t* test using Minitab^®^18 software package (Minitab Inc., State College, PA, USA).

## Results

### ddRAD-seq based genotyping and SNP discovery

**Fig. 1:**
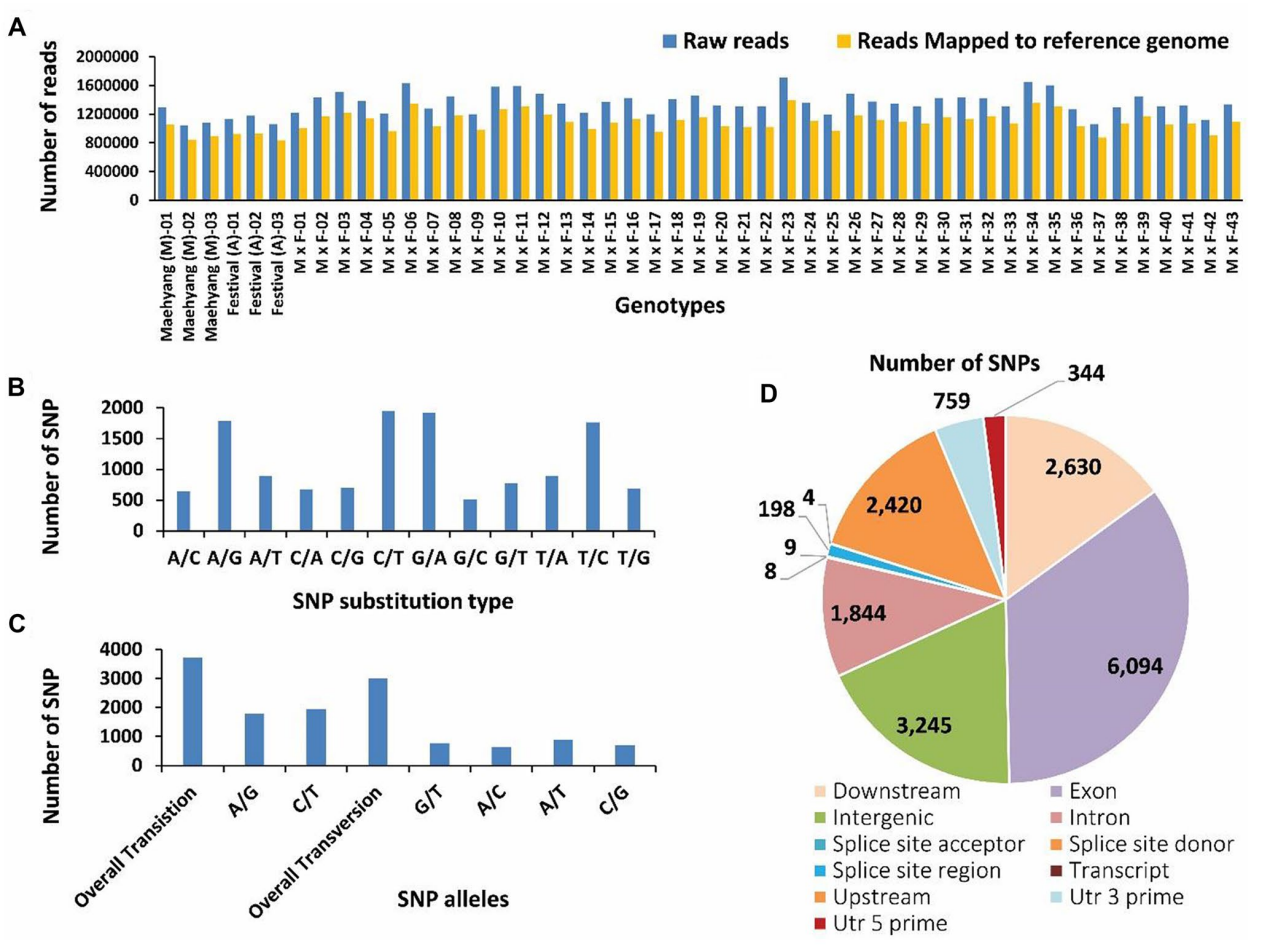
Graphs showing the frequency of raw reads mapped on the reference genome of *Fragaria* × *ananassa* (**a**), frequency of substitution (**b**) and transition/transversion (**c**) type SNP variants, and various SNP categories (**d**) identified by ddRAD-sequencing of the strawberry cultivars, Maehyang (♀) and Festival (♂), and their F1 offspring

The genomic DNA of strawberry genotypes were double digested with *PstI* and *MspI* restriction enzymes to generate ddRAD-seq representation libraries which were subsequently sequenced using Illumina HiSeq 2000 platform. The paired-end sequencing of individuals yielded a total of 65,915,564 reads (total 10.31 GB sequenced data) with an average of 1,345,212 (~ 1.3 million) reads per accessions. A total of 26,655,685 high quality reads were retained and an average of 80.8% of total reads (which ranged from 77.6 to 82.6%) were aligned against *Fragaria* × *ananassa* genome version FAN_r1.1 (Fig. 1a). The detailed statistics of raw-, cleaned-, and mapped-reads, and genotype-wise alignment ratio are summarized in Table S1. Finally, based on strict SNP filtration criteria (see materials and methods section), a total of 12,698 high quality SNPs were retained from the mapped reads.

### Distribution patterns of identified SNPs

Variable distribution of different types of SNPs were observed (Fig. 1b). Among the 12,698 high quality SNP genotypes, the highest proportion of The SNP variant was C/T (1941) followed by G/A (1914), A/G (1787) and T/C (1765). The lowest proportion of SNP genotype was G/C (508) followed by A/C and C/A (642 and 678, respectively) (Fig. 1b). Out of 12,698 SNP genotypes, 3626 (28.5%) and 2790 (21.9%) SNPs were classified as transitions (A/G or C/T) and transversions (G/T, A/C, A/T, or C/G), respectively (Fig. 1c). In general, occurrence of nucleotide transition type SNPs were higher than transversion types (with a transition/tranversion (TS/TV) ratio of 1.30). This could be due to the interchange between purine and pyrimidine nucleotide bases. SnpEff tool version 4.11 was used to annotate the high quality SNPs based on their functional classes (Fig. 1d) which revealed the majority of SNPs (6094; 47.99%) are exonic, followed by 3245 (25.56%) intergenic SNPs. A total of 2630 (20.71%) and 2420 (19.06%) SNPs were located in 1 kb downstream and upstream regions, respectively, while 1844 (14.52%) SNPs were located in intronic regions (Fig. 1d).

### Construction of high density linkage map

**Fig. 2:**
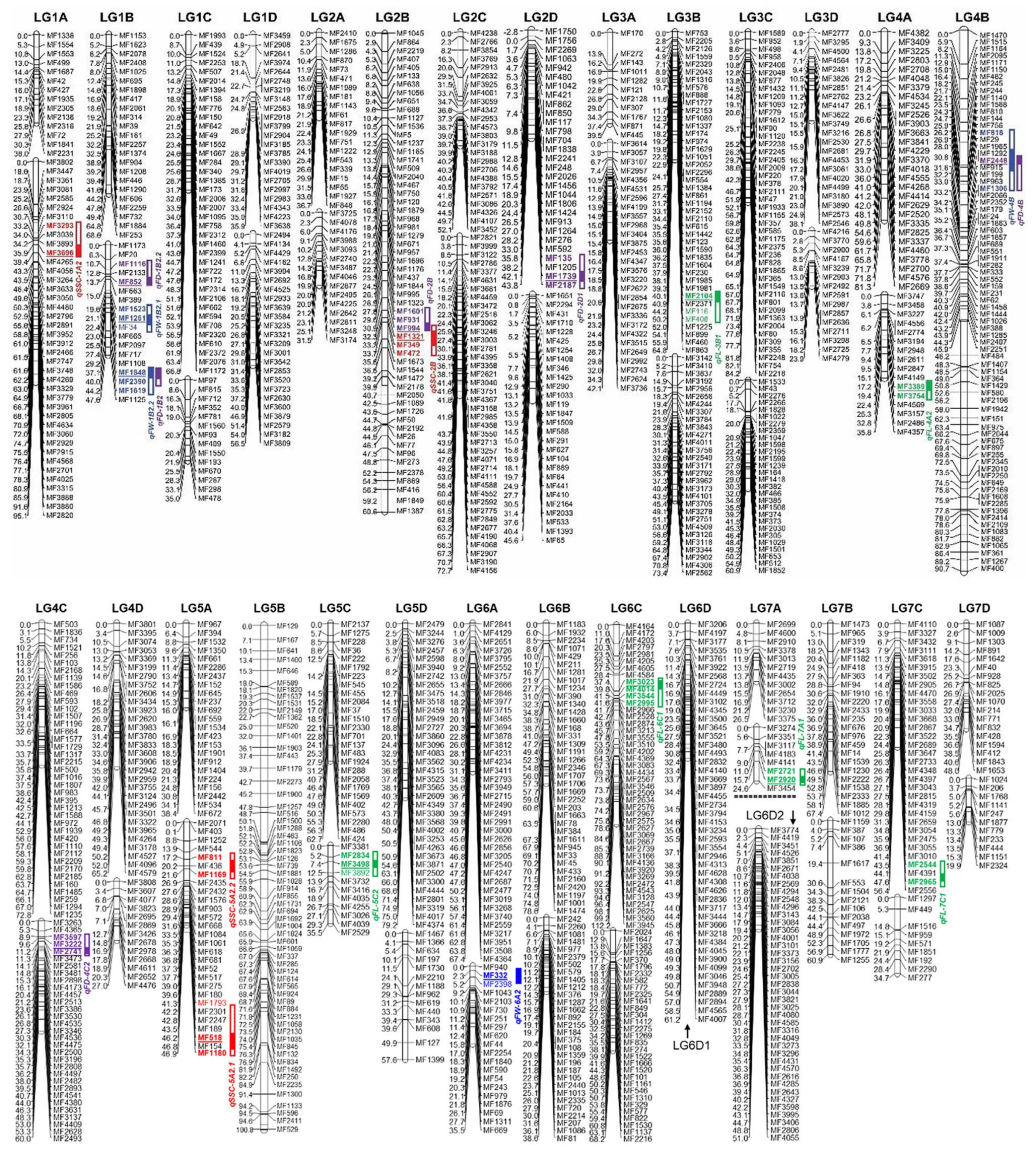
A SNP-based linkage map of *Fragaria* × *ananassa* mapping population derived from the progeny of the cross ‘Maehynag’ (♀) × ‘Festival’ (♂) comprising of 1554 SNP markers. The genetic distances are given in centi-Morgans (cM) in the left each linkage group. The green, purple, blue and red bars (in the right side) indicate the QTLs identified for length (*qFL*), diameter (*qFD*), weight (*qFW*) and soluble sugar content (*qSSC*) of strawberry fruits, respectively. The details of the linkage groups are shown in Table 1 and Tables S3; and the details of the QTLs are shown in Table 2. Underlined markers indicate markers located at peak LOD position

Among 12,698 high quality SNPs, 4638 fitted our selection criteria of polymorphism between the parents i.e., presence of heterozygous loci at least in 1 parent. Out of 4638 markers, 207 markers that had a minimum of 10% missing data in the population and 1165 markers that showed significant distortion from expected ratio of Mendelian segregation were excluded in the subsequent analysis. From the 3266 markers showing significant Mendelian segregation, 1332 pairs (consisting of 1139 unique markers) showed exactly identical patterns of segregation (Table S2). Finally, 53 linkage groups were selected based on the minimum LOD threshold of 5.0 where 1554 SNP markers were mapped that spanned a total genetic distance of 2937.93 cM representing all 28 chromosomes of *Fragaria* × *ananassa* (Table 1; Fig. 2). The markers that were not mapped within these 53 LGs were either mapped in smaller groups or not linked to any of the recognized LGs or showed conflicting segregation pattern with other markers of the same linkage group at the selected LOD threshold. The average length of the linkage groups was 56.12 cM that ranged between 19.89 cM (LG7D2) and 120.27 cM (LG6C1). A minimum of eight and a maximum of 90 markers were mapped in linkage group LG7A2 (24.62 cM) and LG5B (94.5 cM), respectively; with an average of 29.30 markers per linkage group.

**Table 1:** Summary statistics of the linkage groups constructed using the population arising from the cross of cultivated strawberry cultivars ‘Maehyang’ × ‘Festival’ along with their physical span on the wild strawberry (*F. vesca*) genome (v4.0.a1)

SL	Linkage group	Length (cM)	Total no. of mapped marker	First and last mapped marker	Average interval (cM/locus)	Longest gap (cM)	*F. vesca* chromo-some	LG physical start (bp)^a^	LG physical end (bp)^b^	Physical Span (bp)^c^
1	LG1A1	38.02	14	MF1336-MF2231	2.72	7.771	Fvb1	112,982	11,146,492	11,033,510
2	LG1A2	95.14	40	MF3802-MF2820	2.38	18.61	Fvb1	144,009	30,380,107	30,236,098
3	LG1B1	68.61	23	MF1153-MF253	2.98	15.37	Fvb1	387,386	21,918,298	21,530,912
4	LG1B2	47.63	18	MF1173-MF1125	2.65	7.938	Fvb1	1,673,692	35,339,909	33,666,217
5	LG1C1	66.79	38	MF1993-MF1172	1.76	8.66	Fvb1	352,710	22,983,007	22,630,297
6	LG1C2	34.96	14	MF97-MF478	2.50	8.63	Fvb1	220,039	20,650,547	20,430,508
7	LG1D1	35.89	22	MF3459-MF3638	1.63	13.54	Fvb1	387,386	23,532,684	23,145,298
8	LG1D2	56.45	24	MF2494-MF3809	2.35	6.86	Fvb1	7,438,667	21,999,984	14,561,317
9	LG2A1	36.68	20	MF2410-MF848	1.83	5.36	Fvb2	804,175	8,552,409	7,748,234
10	LG2A2	31.47	15	MF3725-MF3174	2.10	6.37	Fvb2	391,734	30,170,922	29,779,188
11	LG2B	60.59	59	MF1045-MF1387	1.03	3.75	Fvb2	1,841,390	21,472,695	19,631,305
12	LG2C1	52.08	24	MF4238-MF3452	2.17	8.87	Fvb2	9,714,170	22,307,546	12,593,376
13	LG2C2	72.67	40	MF2821-MF4156	1.82	4.68	Fvb2	11,118,654	29,150,635	18,031,981
14	LG2D1	43.78	28	MF1756-MF2187	1.56	4.83	Fvb2	19,045,498	29,311,308	10,265,810
15	LG2D2	45.57	28	MF1651-MF65	1.63	6.19	Fvb2	6,445,244	29,260,319	22,815,075
16	LG3A1	39.49	11	MF170-MF445	3.59	13.90	Fvb3	259,640	3,706,811	3,447,171
17	LG3A2	42.09	28	MF3614-MF3736	1.50	7.79	Fvb3	24,675	10,007,601	9,982,926
18	LG3B1	64.83	42	MF753-MF863	1.54	8.89	Fvb3	9,806,200	36,165,058	26,358,858
19	LG3B2	73.35	29	MF3142-MF2562	2.53	7.26	Fvb3	3,610,045	30,520,273	26,910,228
20	LG3C1	102.15	64	MF2381-MF1656	1.60	6.92	Fvb3	2,459,989	38,187,038	35,727,049
21	LG3C2	60.87	28	MF1533-MF1852	2.17	12.50	Fvb3	1,747,234	37,730,760	35,983,526
22	LG3D1	38.11	22	MF2777-MF2546	1.73	13.16	Fvb3	33,191,108	38,204,595	5,013,487
23	LG3D2	23.86	15	MF4216-MF4279	1.59	5.63	Fvb3	33,245,210	38,172,850	4,927,640
24	LG4A1	81.34	29	MF4382-MF2669	2.81	9.5	Fvb4	1,119,936	22,258,587	21,138,651
25	LG4A2	35.82	16	MF3747-MF4357	2.24	7.58	Fvb4	1,370,012	19,515,040	18,145,028
26	LG4B	90.68	76	MF1470-MF400	1.19	5.87	Fvb4	344,401	32,978,302	32,633,901
27	LG4C1	72.76	39	MF503-MF1235	1.87	8.80	Fvb4	5,435,337	29,669,545	24,234,208
28	LG4C2	60.03	31	MF3263-MF2493	1.94	6.76	Fvb4	16,581,222	33,847,926	17,266,704
29	LG4D1	65.19	30	MF3801-MF4579	2.17	13.15	Fvb4	23,366,617	33,622,414	10,255,797
30	LG4D2	27.03	13	MF3808-MF4476	2.08	6.78	Fvb4	30,021,667	33,019,392	2,997,725
31	LG5A1	38.39	23	MF967-MF672	1.67	6.36	Fvb5	1,553,408	6,059,192	4,505,784
32	LG5A2	46.89	28	MF2071-MF1180	1.74	8.64	Fvb5	918,028	33,451,634	32,533,606
33	LG5B	94.50	90	MF129-MF529	1.05	6.31	Fvb5	130,730	29,367,998	29,237,268
34	LG5C1	62.67	26	MF2137-MF424	2.41	9.47	Fvb5	412,107	30,286,252	29,874,145
35	LG5C2	35.51	11	MF3381-MF2529	3.23	7.84	Fvb5	6,888,616	19,385,376	12,496,760
36	LG5D1	79.18	36	MF2479-MF4374	2.20	8.56	Fvb5	5,623,131	28,979,152	23,356,021
37	LG5D2	57.57	14	MF1467-MF1399	4.11	12.58	Fvb5	12,505,941	28,038,301	15,532,360
38	LG6A1	67.36	40	MF2841-MF4364	1.68	4.85	Fvb6	59,229	20,835,306	20,776,077
39	LG6A2	35.52	20	MF940-MF669	1.78	7.79	Fvb6	1,682,732	7,841,866	6,159,134
40	LG6B1	78.60	37	MF1183-MF1474	2.12	6.63	Fvb6	3,657,849	26,798,353	23,140,504
41	LG6B2	38.63	30	MF242-MF81	1.29	4.46	Fvb6	1,981,976	31,678,581	29,696,605
42	LG6C1	120.27	72	MF4164-MF3945	1.67	10.94	Fvb6	667,185	36,710,622	36,043,437
43	LG6C2	68.21	31	MF2024-MF2216	2.20	12.90	Fvb6	978,324	35,962,768	34,984,444
44	LG6D1	61.17	47	MF3206-MF4007	1.30	4.55	Fvb6	268,044	39,453,033	39,184,989
45	LG6D2	51.05	41	MF3774-MF4055	1.25	4.14	Fvb6	139,677	39,197,465	39,057,788
46	LG7A1	26.48	12	MF2699-MF3375	2.21	4.75	Fvb7	2,224,693	7,521,663	5,296,970
47	LG7A2	24.62	8	MF3274-MF3454	3.08	8.93	Fvb7	1,443,298	15,126,833	13,683,535
48	LG7B1	67.36	23	MF1473-MF1012	2.93	9.22	Fvb7	1,123,756	20,064,741	18,940,985
49	LG7B2	60.87	15	MF1159-MF1255	4.06	12.02	Fvb7	4,644,080	17,895,814	13,251,734
50	LG7C1	56.43	32	MF4110-MF2556	1.76	8.85	Fvb7	5,168,144	20,476,274	15,308,130
51	LG7C2	34.72	9	MF1297-MF277	3.86	9.52	Fvb7	21,052,569	24,102,270	3,049,701
52	LG7D1	48.04	18	MF1087-MF1653	2.67	7.43	Fvb7	19,272,181	24,078,363	4,806,182
53	LG7D2	19.89	11	MF1024-MF2324	1.81	7.38	Fvb7	22,566,455	24,095,016	1,528,561
Total	n/a	2937.93	1554	n/a	2.14	446.28	n/a	n/a	n/a	231.27 Mbp

^a,b^The overall physical start and end positions of all markers of one linkage group in the *F.vesca* genome v4.0.a1

^c^The distance between the LG physical start and LG physical end that indicates the overall physical span of all the markers of one linkage group in a particular chromosome of the *F.vesca* genme v4.0.a1

The map resolution corresponded to an average marker density of 2.14 cM per locus with the longest marker interval of 18.61 cM being observed in LG1A2. The sequence tags corresponding to the SNP markers were mapped on the *F. vesca* genome v4.0.a1 (one of the four sub-genomes of the cultivated octoploid *Fragaria* × *ananassa*), which revealed a total coverage of 231.27 Mbp (96.36%) of the diploid *F. vesca* genome (240 Mb). In addition, the map distances of the SNP markers and their corresponding physical positions on the *F. vesca* chromosomes showed high degree of collinearity, except for few less dense and shorter linkage groups such as LG1A1, LG1B2, LG1C2, LG2A1, LG2A2 and LG7A2 (Fig. S1). The details of the linkage groups, marker density and marker distance along with their corresponding physical positions on *F. vesca* genome are shown in Table 1 and Table S3. The SNP positions and SNP alleles in all genotypes along with their corresponding gene IDs are shown in Table S4.

### Phenotypic assessment

**Fig. 3:**
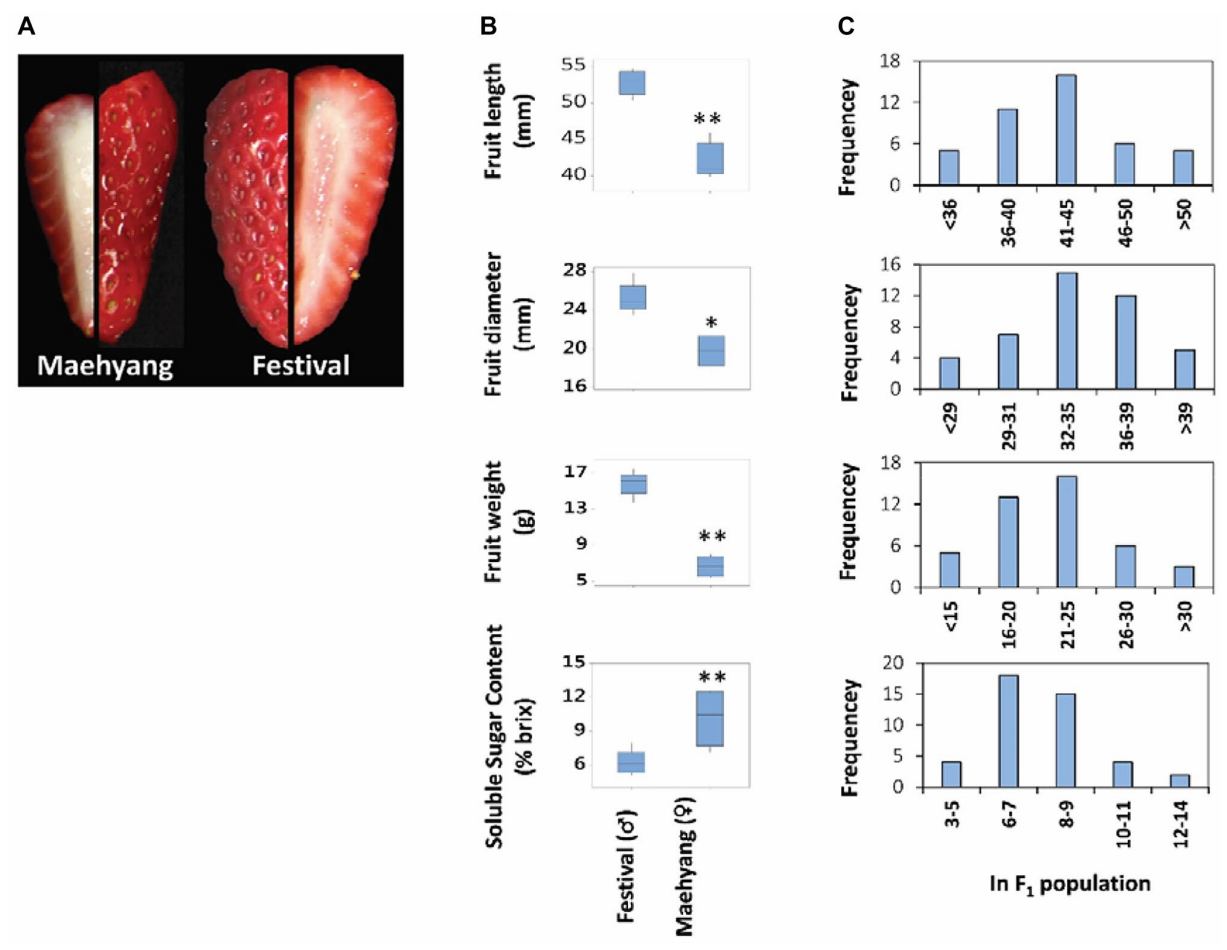
Fruit phenotypes (**a**), differences in length, diameter, weight and sugar content of fruits (**b**) of high- and low-sugar containing cultivars, Maehyang (♀) and Festival (♂), and their frequency distribution in F_1_ population (**c**). Phenotypic data in parents are presented as mean ± SE (*n* = 5). Asterisks * and ** represent significant differences between the parental cultivars at *p* < 0.05 and *p* < 0.01, respectively, by Student’s *t* test

Significant (*p* < 0.05) differences were observed for the length, diameter, weight and soluble sugar content of the fruits of two parental cultivars, ‘Maehyang’ and ‘Festival’ (Fig. 3). The traits FL, FD and FW of ‘Festival’ were higher (Fig. 3) while the SSC of this cultivar (6.17°Bx) was significantly lower compared to that of ‘Maehyang’ (10.13°Bx). The crossing population raised from these two parents showed normal distribution of the traits with FL ranging from 33.81 to 55.60 mm, FD ranging from 26.36 to 70.14 mm, FW ranging from 10.45 to 33.75 g and SSC ranging from 4.90 to 13.10°Bx (Fig. 3). Among these, extreme phenotypes in F_1_ plants, indicating transgressive segregation, is observed for FD, FW and SSC. Correlation analysis of the strawberry fruit quality traits among the F_1_ progeny indicates that fruit SSC was significantly negatively correlated with fruit length and fruit weight. However, the correlation between fruit SSC and fruit diameter was not significant (Table S5).

**Table 2:** QTLs detected for length, diameter, weight and soluble sugar content of strawberry fruits in ‘Maehyang’ × ‘Festival’ population based on the composite interval mapping (CIM) analysis of the ddRAD-seq derived genome-wide SNPs

QTL Name	Linkage group	Marker	Genetic distance (cM)^a^	LOD thre-shold^b^	LOD Max^c^	Addi-tive effect^d^	R^2e^	*Fragaria* × *ananassa* genome ID	*F. vesca* Physical position (bp)
*QTLs for fruit length*									
qFL-6C1*	LG6C1	MF3023-MF2522	37.26–40.66	2.3	10.58	− 15.9	0.24	FAN_iscf00104879.1–FAN_iscf00160810.1	Fvb6:27,319,197–32,065,711
***qFL-7C1***	LG7C1	MF2965-MF2544	43.5–47.6	2	9.96	10.51	0.28	FAN_iscf00037535.1–FAN_iscf00229100.1	Fvb7:15,883,769–15,884,248
***qFL-3B1***	LG3B1	**MF2104-**MF408	40.077–50.223	2.1	8	− 17.5	0.31	FAN_icon19954809.1–FAN_iscf00387732.1	Fvb3:11,547,492–17,768,445
***qFL-4A2***	LG4A2	**MF3389-**MF3754	17.194–19.368	2.2	5.09	19.03	0.35	FAN_icon19840611.1−FAN_iscf00160711.1	Fvb4:12,462,307–14,470,294
qFL-5C2*	*LG5C2*	**MF3498**-MF2834	5.219–7.428	2.1	4.52	− 8.21	0.26	FAN_iscf00088923.1−FAN_iscf00331071.1	Fvb5:17,328,674–19,385,376
***qFL-7A2***	*LG7A2*	MF2721-**MF2920**	11.018–15.691	2	4.3	− 10.6	0.31	FAN_icon19998173.1−FAN_iscf00008836.1	Fvb7:1,443,298–15,126,833
*QTLs for fruit diameter*									
***qFD-4C2***	*LG4C2*	**MF2741**-MF3597	8.853–11.178	1.7	7.9	− 31.7	0.42	FAN_iscf00078828.1−FAN_iscf00051427.1	Fvb4:29,118,956–32,204,721
qFD-2B*	LG2B	MF1601-**MF994**	27.815–30.857	1.6	3.35	9.065	0.16	FAN_iscf00275054.1−FAN_iscf00135334.1	Fvb2:8,447,300–9,035,947
***qFD-1B2***	LG1B2	**MF1848**-MF2390	37.096–39.95	1.7	3.94	16.98	0.36	FAN_iscf00346482.1−FAN_iscf00019693.1	Fvb1:4,034,171–4,207,114
***qFD-4B***	*LG4B*	**MF2448**-MF1306	30.84–33.173	1.7	3.38	− 7.56	0.21	FAN_icon20620372.1−FAN_iscf00244831.1	Fvb4:26,221,362–27,478,921
*QTLs for fruit weight*									
***qFW-1B2.1***	*LG1B2*	MF34-**MF1261**-MF1523	19.6–22.4	2.8	8.37	− 12.3	0.54	FAN_iscf00128046.1–FAN_iscf00150808.1	Fvb1:4,331,273–4,893,189
qFW-1B2.2*	*LG1B2*	**MF1848**-F1619	37.1–44.2	2.8	4.8	8.617	0.24	FAN_iscf00290643.1−FAN_iscf00019693.1	Fvb1:1,673,692–4,034,810
***qFW-4B***	LG4B	MF818-MF1306	26.2–33.17	2.7	4.3	− 7.02	0.3	FAN_iscf00244831.1−FAN_iscf00345769.1	Fvb4:24,382,100–30,792,729
***qFW-6A2***	LG6A2	**MF332-**MF2398	2.32–5.21	2.8	4.07	− 8.07	0.24	FAN_iscf00266073.1−FAN_iscf00355876.1	Fvb6: 6,703,654–7,841,866
*QTLs for fruit soluble sugar content*									
qSSC-1A2*	LG1A2	MF3293-**MF3696**	33.20–35.87	2.6	3	− 2.03	0.25	FAN_iscf00062613.1−FAN_iscf00100711.1	Fvb1:7,154,055–7,645,022
***qSSC-2B***	LG2B	MF994-**MF1321**-MF472	30.86–33.74	2.6	4.6	4.244	0.41	FAN_iscf00135334.1−FAN_iscf00049825.1	Fvb2: 4,096,188–9,035,947
***qSSC-5A2.1***	LG5A2	MF1180-MF154-**MF518**	41.29–46.90	2.7	7.34	3.328	0.5	FAN_icon20112375.1−FAN_iscf00158336.1	Fvb5:3,029,884–8,492,943
***qSSC-5A2.2***	LG5A2	MF811-MF1169	17.17–21.58	2.7	4.29	2.169	0.23	FAN_iscf00290095.1−FAN_icon19417585.1	Fvb5: 9,084,155–918,334

*Asterisk (*) indicate QTLs not detected by multi-locus mixed linear models (mrMLM, pLARmEB and ISIS EM-BLASSO method)

^a^The distance of the QTL in cM (expressed in Kosambi) from the top of the linkage group

^b^The significant threshold logarithm of the odds (LOD) score calculated by the composite interval mapping using Kosambi's map function with 1000 permutation (*p* ≤ 0.05)

^c^The peak LOD score

^d^Positive values indicate QTL alleles were contributed by the parent Maehyang whereas negative values were contributed by the parent Festival

^e^Bold texted markers indicate markers located at LOD peak position

### Identification of QTLs

Composite interval mapping in the ‘Maehyang’ × ‘Festival’ population identified six QTLs for FL and four QTLs for each of FD, FW and SSC (Fig. 2; Table 2). Among the six QTLs for FL, four were negative effect- and two were positive effect-QTLs, respectively, which explained 24–38% of phenotypic variation (*R*^2^). Among the four QTLs for FD, the negative effect QTL *qFD-4C2* on LG4C2, flanked by markers MF2741-MF3597, had the highest LOD score of 7.9 (*p* < 0.05) and explained the highest proportion (42%) of phenotypic variation (*R*^2^). FW related four QTLs explained 24–54% of phenotypic variation, with the QTL *qFW-1B2.1* (within MF34-MF1523 makers) having the highest LOD score (8.37) and the highest proportion of phenotypic variance (54%). Among the four QTLs for fruit SSC, three QTLs namely, *qSSC-5A2.1*, *qSSC-2B* and *qSSC-5A2.*2 located on linkage groups LG5A2, LG2B and LG5A2, respectively, were positive effect QTLs while the QTL *qSSC-1A2* located on LG1A2 was negative effect QTL. Among these QTLs, *qSSC-5A2.1* (flanked by MF1180 and MF518) had the highest LOD score (7.34) and explained the highest proportion of phenotypic variation (50%) among the ‘Maehyang’ × ‘Festival’ population. Several multi-locus mixed linear models such as mrMLM, pLARmEB and ISIS EM-BLASSO were also employed that validated most of the QTLs identified by CIM methods except *qFL-6C1* and *qFL-5C2* for FL, *qFD-2B* for FD, *qFW-1B2.2* for FW, and *qSSC-1A2* for SSC that were not detected by multi-locus mixed linear models (Table 2).

QTLs for FD and FW were co-localized in two linkage groups such as *qFW-1B2.2* and *qFD-1B2.1* on LG1B and *qFW-4D* and *qFD-4B* on LG4B. The former linkage group hosted two more QTLs namely, *qFD-1B2.2* for FD and *qFW-1B2.1* for FW. Sugar related QTL *qSSC-2B* were very closely located with FD related QTL *qFD-2B* on LG2B (Fig. 2). All other QTLs were solitary i.e., they were located on individual linkage group where no other QTLs were located (Fig. 2; Table 2).

### Potential candidate genes within QTL regions

The genes that lie within the flanking regions of the identified QTLs (Table 2) were extracted from the corresponding regions of *Fragaria vesca* genome v4.0.a1. Altogether, 2348 genes were found within the regions of FL related QTLs (Table S6). The marker MF3023 of QTL *qFL-6C1* corresponded to the *Fragaria* × *ananassa* gene FAN_iscf00104879.1 encoding ‘Transcription initiation factor TFIID subunit 12’ and the flanking marker MF2104 QTL of *qFL-3B1* corresponded to gene FAN_iscf00387732.1 encoding ‘Serine/arginine repetitive matrix protein 1’. Within FD and FW related QTL regions, a total of 806 and 1629 genes were found, respectively. Both the flanking markers, MF1601 (FAN_iscf00275054.1) and MF994 (FAN_iscf00135334.1) of fruit diameter related QTL *qFD-2B* encoded for transcription factor genes (Transcription factor TFIIIB component B and Transcriptional activator DEMETER, respectively) and the flanking marker MF2390 of QTL *qFD-1B2* encoded a Translation machinery-associated protein (FAN_iscf00346482.1). The corresponding gene (FAN_iscf00345769.1) of the flanking marker MF818 of FW-related QTL *qFW-4B* encoded a ‘NA-binding protein HEXBP’ and the corresponding gene (FAN_iscf00266073.1) of the flanking marker MF332 of QTL *qFW-6A2* encoded a putative receptor protein kinase *ZmPK1*. These transcription regulation related activity of the linked marker associated genes may have roles in genetic regulation of the corresponding traits.

Within genes of SSC related QTL regions, fourteen genes were identified to be related with sugar biosynthesis. These fourteen genes, particularly UDP-glucose 4-epimerase GEPI48-like gene (FvH4_5g04740.1), UDP-glucose 6-dehydrogenase family protein (FvH4_5g14230.1), Glucose-6-phosphate/phosphate translocator-related protein (FvH4_5g05430.1), along with the gene FAN_iscf00049825.1 hosting the flanking marker MF472 of QTL *qSSC-2B* encoding a UDP-glucosyltransferase gene may have potential roles in sugar biosynthesis in strawberry (Tables S7 and S8). In addition, seven invertase family genes and the transcription factors genes identified within sugar content related QTL regions may also have roles in sugar metabolism.

### Developemnt of markers linked with sugar content

**Fig. 4:**
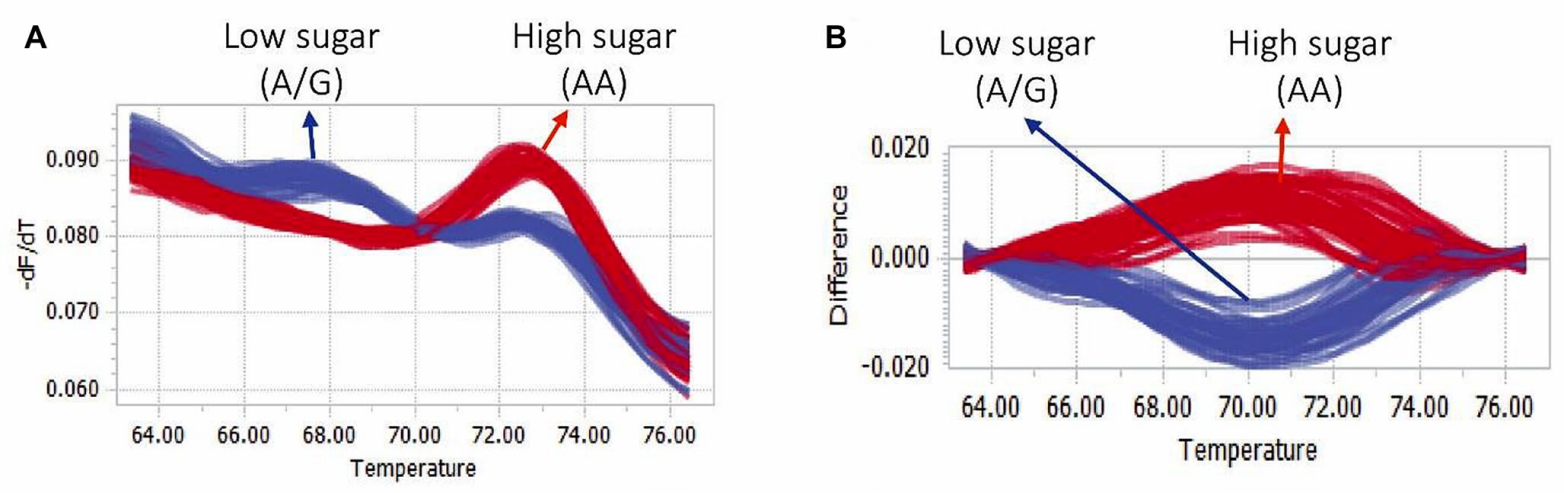
Normalized melting peaks (**a**) and the difference plots (**b**) of the high resolution melting (HRM) analysis of F1 lines and 23 commerial strawberry lines using the developed HRM marker MF154

Four SNP markers (MF518, MF1321, MF1169, and MF3696) located at the peak LOD positions of the corresponding SSC related QTLs and one SNP (marker MF154) on the gene FAN_iscf00021287, a key candidate gene for sugar biosynthesis (Shanmugam et al. 2017) located within the flanking region of QTL* qSSC-5A2.1* were targeted for developing high resolution melting (HRM) marker linked with sugar content in strawberry (Table S9). Among these five HRM markers, reasonable match between the phenotypic and genotypic observations was only found for the marker MF154, designed based on the SNP A1^723^G (for high and low sugar contents, respectively) of the UDP-glucose 4-epimerase GEPI48-like gene FAN_iscf00021287 (Table S9). The prediction accuracy of the marker was 81.39% and 86.95% for the tested 43 F1 individuals and 23 commercial culitvars, respectively (Fig. 4; Table S10). This indicates it’s suitability in marker assisted breeding aiming at improving the trait via molecular breeding. In addition, the allelic influence of this marker on the overall variation in fruit weight in the F_1_ population was evaluated using a general linear model with the “lm” function from the “stats” package in R. We found no significant (*R*^2^ = 0.073, *p* = 0.39) effect of allelic difference on fruit weight (Fig. S2). This indicates that selection for fruit SSC is independent of fruit weight.

## Discussion

This study describes construction of high-density linkage map and detection of QTLs for key fruit quality reated traits using genome-wide SNPs derived ddRAD-seq technique from a mapping population raised from ‘Maehyang’ and ‘Festival’ (M × F). These two cultivars were chosen based on their contrasting characteristics for a number of agronomic and fruit quality traits and for their diverse geographical origin and adaptation. The variety, Maehyang is a relatively new Korean cultivar originated from ‘Tochinomine’ × ‘Akihime’ and is highly valued for its vigorous and erect type growth, weak dormancy, long, conical and red colored fruit, high sugar content, resistance to powdery mildew and relatively high marketable yield (Kim et al. 2004). ‘Festival’, on the other hand, is a US patented variety developed from hand-pollinated cross of ‘Rosa Linda’ and ‘Oso Grande’ and is characterized by profuse runnering habit, large calyces, long fruit pedicel, firm fleshed, flavorful, deep red, large fruits having soluble sugar content comparatively less than that of ‘Maehyang’(Chandler 2004).

### Transgressive segregation of fruit quality traits

Transgressive segregations were observed for diameter and weight of fruits in F_1_ population. Such transgressive segregations have been reported in intra-specific levels such as *Oryza sativa*, *Cucumis melo*, *Lolium perenne* and *Pisum sativum* etc. (Mao et al. 2011); in inter-specific level such as *Oryza*, *Prunus* and *Solanum* etc. (Gutiérrez et al. 2010; Quilot et al. 2004) and in polyploids such as *Triticum durum *(Maccaferri et al. 2011) and Saccharum (Ming et al. 2001). The allelic combinations of the genetically distant parents having complementary gene action could possibly be a reason behind such extreme F_1_ phenotypes in our study. Besides, potential epistatic interactions between the multiple alleles of this high-ploidy species may contribute to the dominance or over-dominance effect which results in such transgressive segregants (Coelho et al. 2007; Flagel and Wendel 2009). Nonetheless, such transgressive phenotypic expressions in crossing population of this species could be exploited for developing high performing varieties.

### Linkage map of ‘*Maehyang*’ × ‘*Festival*’ population

The constructed 53 LGs spanned a total genetic distance of 2937.93 cM with an average marker density of 2.14 cM per locus in M × F population. This marker density and physical span is greater than the previously reported linkage maps (Isobe et al. 2013; Lerceteau-Köhler et al. 2003; Rousseau-Gueutin et al. 2008; Weebadde et al. 2008; Sargent et al. 2009; Zorrilla-Fontanesi et al. 2011; Castro et al. 2015; Dijk et al. 2014). Among the SNP marker based linkage groups, Davik et al. (2015) mapped 907 markers in 31 linkage groups which spanned 1581.5 cM in 145 F_1_ hybrid of Sonata (♀) × Babette (♂) population that is estimated to resolve 79% of *Fragaria* × *ananassa* genome. In the linkage map of ‘232’ × ‘1392’ population, Sánchez-Sevilla et al. (2015) mapped 2089 SNP markers in 33 LGs which spanned a total of 2489.56 cM. Recently, Vining et al. (2017) mapped 1163 SNPs in the ‘Tribute (29 LG)’ × ‘Honeoye (33 LG)’ population, 2136 SNPs in ‘Holiday (38 LG)’ × ‘Korona (49 LG)’ population and the highest 3,912 SNPs in ‘Redgauntlet (39 LG)’ × ‘Hapil (37 LG)’ population. The higher number of mapped SNPs in the latter population could be attributed to the fact that SNPs were detected by aligning the raw reads against *F. vesca* reference genome directly, instead of *Fragaria* × *ananassa* genome. Whereas SNPs of our work was detected by aligning the raw reads against *Fragaria* × *ananassa* reference genome (FAN_r1.1). The total coverage of 96.36% of the latest diploid *F. vesca* genome by the mapped markers and high degree of collinearity between the LG-wise markers along the length of the corresponding *F. vesca* chromosomes further indicate towards the robustness of the constructed linkage map. The constructed linkage maps, thus may assist in future QTL mapping for various important traits in different genetic backgrounds and agro-ecological environments.

### QTLs for fruit growth and quality traits

Altogether eighteen QTLs; six for FL and four for FD, FW and SSC each were identified in the M × F population (Fig. 2; Table 2). Most of the QTLs were also detected by the multi-locus mixed linear models such as mrMLM, pLARmEB and ISIS EM-BLASSO (Tamba et al. 2017; Wang et al. 2016; Wen et al. 2018; Zhang et al. 2017). For FL, Lerceteau-Köhler et al. (2012) identified similar number of QTLs for FD. For FW, however, Zorrilla-Fontanesi et al. (2011) identified two and Lerceteau-Köhler et al. (2012) identified three QTLs in ‘232’ × ‘1392’ and ‘Capitola’ × ‘CF1116’ populations, respectively. Compared to our four QTLs for fruit SSC, three QTLs have been identified by three independent studies (Lerceteau-Köhler et al. 2012; Zorrilla-Fontanesi et al. 2011; Castro and Lewers 2016). In these three studies, QTLs were identified based on AFLP, SSR and SCAR markers. The use of few common markers facilitated the comparison of the constructed linkage groups and identified that a number of QTLs were actually located on homeologous linkage groups, suggesting common genetic control of related traits in multiple backgrounds (Pott et al. 2000). The QTLs of this study were, however, based on genome-wide SNP based markers, thus could not be directly compared with those previous reports.

QTLs detected for total SSC in fruits in this study are fewer compared to the cumulative number of previously identified QTLs for individual sugar compounds such as sucrose (2), glucose (5) and fructose (2) by Lerceteau-Köhler et al. (2012). The higher proportion of phenotypic variations (23–50%) explained by the QTLs for SSC, in contrast to the smaller proportions of phenotypic variations explained by the QTLs for individual sugar components, provides a possible explanation for this. Besides SSC, the titratable acidity (TA) and their delicate ratio determines the flavor of strawberry fruits, requiring the selection of fruits that have suitable balance between sweetness and tartness. Several QTLs for TA and SSC/TA has previously been reported to be collocated with the QTLs for SSC (Lerceteau-Köhler et al. 2012; Zorrilla-Fontanesi et al. 2011; Castro and Lewers 2016; Vallarino and Pott 2019). However, the significant but low correlation co-efficient value between these two traits suggested the possibility of independent selection of each of the traits (Castro and Lewers 2016). Overall, identification of several QTLs for a particular trait, each explaining a small individual proportion of the total phenotypic variation indicates complex quantitative inheritance and multi-loci control of these traits (Lerceteau-Köhler et al. 2012; Pott et al. 2000; Paran and Knaap 2007).

Correlation analysis indicated that fruit length is positively correlated with fruit weight, and both these attributes are negatively correlated with fruit SSC (Table S5). This has practical breeding implication in the sense that selection for one trait might negatively affect the selection of another trait. Careful consideration of this point will be necessary for breeding schemes aiming at improving fruit size and fruit sugar contents.

### Distribution of QTLs within the linkage map

Among the 53 linkage groups, only 14 hosted all the 18 detected QTLs with LG1B2 hosting maximum four, and LG2B, LG4B and LG5A2 hosting two QTLs each (Fig. 2). Moreover, QTLs for FD and FW co-localized in two linkage groups such as QTLs *qFD-1B2.1* and *qFW-1B2.2* on LG1B2, and QTLs *qFD-4B* and *qFW-4D* on LG4B. The high and significant correlation between these traits (*R* = 0.512, *p* value = 0.003) in the F_1_ population also indicates potential control by the common genetic regions, possibly by same or tightly linked genes (Table S5). Besides the two co-located QTLs, LG1B2 hosts two more QTLs, *qFW-1B2.1* for FW and *qFD-1B2.2* for FD (total four QTLs throughout the length of this LG). This indicates that this chromosomal region may be the key genetic determinants of these fruit growth and size related traits. A previous study has reported one-fourth of fruit quality related QTLs to be clustered in two linkage groups where QTLs for fruit shape, diameter and weight were particularly found to be co-located (Lerceteau-Köhler et al. 2012). In our population, sugar content related QTL, *qSSC-2B* and FD related QTL, *qFD-2B* were found to be very closely located on LG2B, even though no significant correction (*R* = 0.46, *p *value = 0.13) were observed for these two traits.

Such QTL clusters have also been observed in strawberry for anthocyanin content, antioxidant property (Castro and Lewers 2016), fruit numbers, yield and quality traits (Zorrilla-Fontanesi et al. 2011), and for various fruit quality traits in other crops including pepper, tomato and for fiber development related traits in cotton (Ring et al. 2013; Fulton et al. 1997; Saliba-Colombani et al. 2001). QTL clusters, in genetic terms, suggest the intricately regulated gene networks with potential pleotropic effects; while in breeding terms, indicate the possibility of selection for multiple traits, simultaneously.

### Candidate genes for fruit growth and size related traits

The key candidate genes within the QTL regions for fruit size related traits such as length, diameter and weight of fruits include transcription factors (TF), and plant growth and development related genes (Tables S7 and S8). Several closely linked QTL marker genes encoded some important TFs such as FAN_iscf00104879.1—‘Transcription initiation factor TFIID subunit 12’ (marker MF3023 of QTL *qFL-6C1*), FAN_iscf00387732.1—‘Serine/arginine repetitive matrix protein 1’ (marker MF2104 *linked to qFL-3B1*), both the flanking marker genes, FAN_iscf00275054.1—‘Transcription factor TFIIIB component B’ and FAN_iscf00135334.1—‘Transcriptional activator DEMETER’ (flanking markers MF1601 and MF994 of QTL *qFD-2B*) and FAN_iscf00266073.1 – ‘putative receptor protein kinase *ZmPK1*’ (MF332 of QTL *qFW-6A2*), etc.

Auxin play important role in the early stages of strawberry fruit development where the receptacle growth is dependent on auxin delivery from the achenes. Auxin biosynthesis is dependent on tryptophan aminotransferase (TAA and TAR) and flavin-dependent monooxygenases (YUCCA) enzymes (Pattison et al. 2014; Stepanova et al. 2011). In woodland strawberry genome, four tryptophan aminotransferases (TAA) have been identified and in cultivated strawberry, three genes namely, *FaTAA1, FaTAR1,* and *FaTAR2* were expressed in fruits (Estrada-Johnson et al. 2017). In our population, several tryptophan aminotransferase genes including *FaTAA1* (FvH4_4g25850.1) and *FaTAA2* (FvH4_7g02760.1) were found within FW related QTL *qFW-4B* and FL related QTL *qFL-7A1*, respectively. In addition, within the sugar content related QTL *qSSC-5A21*, three tryptophan aminotransferase genes (FvH4_5g05900.1, FvH4_5g05880.1, and FvH4_5g05890.1) were found. Six auxin responsive genes (FvH4_1g09150.1, FvH4_3g21460.1, FvH4_3g23200.1, FvH4_4g22430.1, FvH4_4g23230.1 and FvH4_5g27860.1) and six auxin efflux carrier genes (FvH4_5g26320.1, FvH4_5g26370.1, FvH4_5g26330.1, FvH4_6g35590.1, FvH4_4g32450.1 and FvH4_4g32170.1) were found within FL, FD and FW related QTL regions (Tables S5).

We have identified three cytokinin oxidase/dehydrogenase genes (FvH4_7g02150.1, FvH4_1g07610.1 and FvH4_1g07620.1) within FL related QTL *qFL-7A1*. Cytokinin dehydrogenase genes and gibberellin 2-beta-dioxygenase genes were found to be upregulated in pear fruits whose overexpression is believed to be involved in cytokinin and gibberellin metabolism that largely controls cell division (Jiang et al. 2018) and hence, fruit size. The *NAC, ERF* and *bHLH* transcription factors were highly related with cytokinin dehydrogenase and gibberellin dioxygenase genes (Jiang et al. 2018). The *SlKLUH* gene encoding a P450 enzyme of the CYP78A subfamily within a major QTL (fw3.2) for tomato fruit size/weight is believed to be involved in enlarging fruit volume through an increase in cell number within the pericarp and septum tissues (Azzi et al. 2015; Chakrabarti et al. 2013).

Four genes (FvH4_6g34910.1, FvH4_6g34950.1, FvH4_7g09250.1 and FvH4_7g09260.1) within FL related QTLs, 10 genes (FvH4_4g29160.1, FvH4_4g29370.1, FvH4_4g29410.1, FvH4_4g29420.1, FvH4_4g29430.1, FvH4_4g29440.1, FvH4_4g29450.1, FvH4_4g29480.1, FvH4_4g29490.1 and FvH4_4g29500.1) within FD related QTLs and 16 genes (FvH4_1g03910.1, FvH4_1g08780.1, FvH4_1g08790.1, FvH4_1g08830.1, FvH4_4g22540.1, FvH4_4g22550.1, FvH4_4g29160.1, FvH4_4g29370.1, FvH4_4g29410.1, FvH4_4g29420.1, FvH4_4g29430.1, FvH4_4g29440.1, FvH4_4g29450.1, FvH4_4g29480.1, FvH4_4g29490.1 and FvH4_4g29500.1) within FW related QTLs encoding for P450 enzyme were found in our study.

### Candidate genes and molecular marker for sugar content

A total of 2007 genes were found within the SSC related QTL regions of which 13 genes may have role in sugar metabolism (Tables S6 and S8). The flanking marker MF472 of QTL *qSSC-2B* is found within the UDP-glucosyltransferase gene (FAN_iscf00049825.1). One glucose-6-phosphate gene (FANhyb_rscf00000021.1.g00018.1—*FaGlu8*) in addition to ‘UDP-glucose 6-dehydrogenase’ and ‘UDP-glucose 4-epimerase GEPI48-like gene’ (FANhyb_rscf00000538.1.g00003.1- *FaGlu15*) were very recently shown to be upregulated in ripe fruits of high sugar cultivars, Shanmugam et al. (2017) and one ‘glucose 6-phosphate/phosphate translocator 1’ gene (*FaGPT1*) were also recently found to be expressed in mature fruits of high sugar cultivars, Geumsil, Aram, Maehyang and Okmae (Lee et al. 2018). The flanking marker MF472 of QTL *qSSC-2B* is harbored in ‘UDP-glucosyltransferase gene’ (FAN_iscf00049825.1) which may have direct roles in sugar biosynthesis (Tables S7 and S8).

The invertases irreversibly hydrolyze sucrose to glucose and fructose and thus play crucial roles in carbohydrate partitioning and plant development (Chen et al. 2015). Three invertase genes namely, *FaINVG-1, FaINVA-3* and *FacwINV2-1* were recently found to show higher expressions in high sugar-content cultivars (Lee et al. 2018). However, none of these are found within our QTL regions. Instead, the seven invertase family genes, of which three are neutral/alkaline invertases, were found within our QTL regions (Tables S7 and S8). These genes may have roles in sugar metabolism.

In addition, a HRM marker (MF154) was developed based on the SNP A1^723^G of the UDP-glucose 4-epimerase GEPI48-like gene FAN_iscf00021287 for selecting high vs low sugar containing genotpes. The gene was located within the QTL *qSSC5A2*.1 on linkage group LG5A2 and was found to be a key gene regulating sugar biosynthesis in progressively ripening strawberry fruits (Shanmugam et al. 2017). Evaluation of the influence of allelic difference of the developed HRM marker (MF154) for fruit SSC on fruit weight in the segregating F_1_ population via general linear model revealed that there is no significant influence of allelic difference of fruit SSC on the fruit weight. This is understandable, since multiple non-collocating QTLs with minor effects were identified for these two traits indicating that different sets of genes, each with minor effects, may be acting on these two traits. In breeding terms, this indicate that selection for fruit SSC is independent of selection for fruit weight in this population.

## Conclusion

This is the first report of QTLs for fruit quality traits using genome-wide SNP based high density linkage map in cultivated strawberry. The detected genome-wide SNPs will supply abundant choice of transferrable markers for future genetic studies. The constructed high density linkage map will serve as reference for precise sequence scaffold anchoring and orientations in this species. Besides the identified QTLs, the linked markers upon validation using larger populations, and in other genetic backgrounds and environments, and the developed HRM marker can be used as mass screening tools in breeding programs. The genes identified within these QTL regions will enhance our understanding of the genetics of these traits and can be targeted for development of varieties with desired fruit quality traits via breeding and biotechnological means.

## Electronic supplementary material

Below is the link to the electronic supplementary material.

Supplementary Materials: The following are available online at www.mdpi.com/xxx/s1 (DOCX 463 kb)

Supplementary file2 (XLSX 44 kb)

Supplementary file3 (XLSX 210 kb)

Supplementary file4 (XLSX 341 kb)

Supplementary file5 (XLS 1115 kb)

## Data Availability

The raw reads generated by ddRAD-seq technique were deposited into Sequence Read Archive (SRA) under NCBI accession PRJNA478299 accessible from https://www.ncbi.nlm.nih.gov/sra/PRJNA478299.

## Abbreviations

AFLP: Amplified Fragment Length Polymorphism
DArT: Diversity Arrays Technology
ddRAD-seq: Double digest restriction-associated DNA sequencing
GBS: Genotyping by sequencing
LG: Linkage group
LOD: Logarithm of odds
SCAR: Sequence characterized amplified regions
SDRF: Single dose restriction fragment
SNP: Single nucleotide polymorphisms
SRA: Sequence Read Archive
SSR: Simple sequence repeats
*M* × *F*: Maehyang (♀) × Festival (♂)

## References

[CR1] Aitken KS, Jackson PA, McIntyre CL (2006). Quantitative trait loci identified for sugar related traits in a sugarcane (Saccharum spp.) cultivar × Saccharum officinarum population. Theor Appl Genet.

[CR2] Alvarez-Suarez JM, Giampieri F, Tulipani S, Casoli T, Di Stefano G, González-Paramás AM, Santos-Buelga C, Busco F, Quiles JL, Cordero MD (2014). One-month strawberry-rich anthocyanin supplementation ameliorates cardiovascular risk, oxidative stress markers and platelet activation in humans. J Nutr Biochem.

[CR3] Azzi L, Deluche C, Gévaudant F, Frangne N, Delmas F, Hernould M, Chevalier C (2015). Fruit growth-related genes in tomato. J Exp Bot.

[CR4] Baird NA, Etter PD, Atwood TS, Currey MC, Shiver AL, Lewis ZA, Selker EU, Cresko WA, Johnson EA (2008). Rapid SNP discovery and genetic mapping using sequenced RAD markers. PLoS ONE.

[CR5] Bassil NV, Davis TM, Zhang H, Ficklin S, Mittmann M, Webster T, Mahoney L, Wood D, Alperin ES, Rosyara UR (2015). Development and preliminary evaluation of a 90 K Axiom^®^ SNP array for the allo-octoploid cultivated strawberry Fragaria × ananassa. BMC Genomics.

[CR6] Browning SR, Browning BL (2007). Rapid and Accurate haplotype phasing and missing-data inference for whole-genome association studies by use of localized haplotype clustering. Am J Hum Genet.

[CR7] Castro P, Lewers KS (2016). Identification of quantitative trait loci (QTL) for fruit-quality traits and number of weeks of flowering in the cultivated strawberry. Mol Breed.

[CR8] Castro P, Bushakra JM, Stewart P, Weebadde CK, Wang D, Hancock JF, Finn CE, Luby JJ, Lewers KS (2015). Genetic mapping of day-neutrality in cultivated strawberry. Mol Breed.

[CR9] Chakrabarti M, Zhang N, Sauvage C, Munos S, Blanca J, Canizares J, Diez MJ, Schneider R, Mazourek M, McClead J (2013). A cytochrome P450 regulates a domestication trait in cultivated tomato. Proc Natl Acad Sci.

[CR10] Chandler CK (2004) ‘Strawberry Festival’ strawberry plant, 1–6.

[CR11] Chen Z, Gao K, Su X, Rao P, An X (2015). Genome-wide identification of the invertase gene family in populus. PLoS ONE.

[CR12] Cingolani P, Platts A, Wang LL, Coon M, Nguyen T, Wang L, Land SJ, Lu X, Ruden DM (2012). A program for annotating and predicting the effects of single nucleotide polymorphisms, SnpEff. Fly (Austin).

[CR13] Coelho CM, Wu S, Li Y, Hunter B, Dante RA, Cui Y, Wu R, Larkins BA (2007). Identification of quantitative trait loci that affect endoreduplication in maize endosperm. Theor Appl Genet.

[CR14] Danecek P, Auton A, Abecasis G, Albers CA, Banks E, DePristo MA, Handsaker RE, Lunter G, Marth GT, Sherry ST, McVean G (2011). The variant call format and VCFtools. Bioinformatics.

[CR15] Davik J, Sargent DJ, Brurberg MB, Lien S, Kent M, Alsheikh M (2015). A ddRAD based linkage map of the cultivated Strawberry, Fragaria xananassa. PLoS ONE.

[CR16] Edger PP, Poorten TJ, VanBuren R, Hardigan MA, Colle M, McKain MR, Smith RD, Teresi SJ, Nelson ADL, Wai CM (2019). Origin and evolution of the octoploid strawberry genome. Nat Genet.

[CR17] Elshire RJ, Glaubitz JC, Sun Q, Poland JA, Kawamoto K, Buckler ES, Mitchell SE (2011). A robust, simple genotyping-by-sequencing (GBS) approach for high diversity species. PLoS ONE.

[CR18] Estrada-Johnson E, Csukasi F, Pizarro CM, Vallarino JG, Kiryakova Y, Vioque A, Brumos J, Medina-Escobar N, Botella MA, Alonso JM (2017). Transcriptomic analysis in strawberry fruits reveals active auxin biosynthesis and signaling in the ripe receptacle. Front Plant Sci.

[CR19] Flagel LE, Wendel JF (2009). Gene duplication and evolutionary novelty in plants. New Phytol.

[CR20] Fulton TM, Beck-Bunn T, Emmatty D, Eshed Y, Lopez J, Petiard V, Uhlig J, Zamir D, Tanksley SD (1997). QTL analysis of an advanced backcross of Lycopersicon peruvianum to the cultivated tomato and comparisons with QTLs found in other wild species. TAG Theor Appl Genet.

[CR21] Gutiérrez A, Carabalí S, Giraldo O, Martínez C, Correa F, Prado G, Tohme J, Lorieux M (2010). Identification of a Rice stripe necrosis virus resistance locus and yield component QTLs using *Oryza sativa* × *O. glaberrima* introgression lines. BMC Plant Biol.

[CR22] Henning SM, Seeram NP, Zhang Y, Li L, Gao K, Lee R-P, Wang DC, Zerlin A, Karp H, Thames G (2010). Strawberry consumption is associated with increased antioxidant capacity in serum. J Med Food.

[CR23] Hirakawa HI, Shirasawa KE, Kosugi SH, Tashiro KO, Nakayama SH, Yamada MA, Kohara MI, Watanabe AK, Kishida YO, Fujishiro TS (2014). Dissection of the octoploid strawberry genome by deep sequencing of the genomes of fragaria species. DNA Res.

[CR24] Hossain MR, Natarajan S, Kim H, Michael D, Jesse I, Lee C, Park J, Nou I (2019). High density linkage map construction and QTL mapping for runner production in allo-octoploid strawberry Fragaria × ananassa based on ddRAD-seq derived SNPs. Sci Rep.

[CR25] Isobe SN, Hirakawa H, Sato S, Maeda F, Ishikawa M, Mori T, Yamamoto Y, Shirasawa K, Kimura M, Fukami M (2013). Construction of an integrated high density simple sequence repeat linkage map in cultivated strawberry (Fragaria x ananassa) and its applicability. DNA Res.

[CR26] Jiang S, An H, Luo J, Wang X, Shi C, Xu F (2018). Comparative analysis of transcriptomes to identify genes associated with fruit size in the early stage of fruit development in Pyrus pyrifolia. Int J Mol Sci.

[CR27] Kim T-I, Jang W-S, Choi J-H, Nam M-H, Kim W-S, Lee S-S (2004). Breeding of Strawberry “Maehyang” for Forcing Culture. Korean J Hortic Sci Technol.

[CR28] Kunihisa M (2011). Studies using DNA markers in Fragaria × ananassa: genetic analysis, genome structure, and cultivar identification. J Jpn Soc Hortic Sci.

[CR29] Langmead B, Salzberg SL (2012). Fast gapped-read alignment with Bowtie 2. Nat Methods.

[CR30] Lee J, Noh HKY, Ran S, Lee MH, Jung J, Kim KPD, Hyeon M, Tae N, Kim I, Hyeran SK (2018). Sugar content and expression of sugar metabolism-related gene in strawberry fruits from various cultivars. Theor Appl Genet.

[CR31] Lerceteau-Köhler E, Guérin G, Laigret F, Denoyes-Rothan B (2003). Characterization of mixed disomic and polysomic inheritance in the octoploid strawberry (Fragaria x ananassa) using AFLP mapping. Theor Appl Genet.

[CR32] Lerceteau-Köhler E, Moing A, Guérin G, Renaud C, Petit A, Rothan C, Denoyes B (2012). Genetic dissection of fruit quality traits in the octoploid cultivated strawberry highlights the role of homoeo-QTL in their control. Theor Appl Genet.

[CR33] Lerceteau-Kohler E, Moing A, Guérin G, Renaud C, Maucourt M, Rolin D, Denoyes-Rothan B (2006). QTL analysis for sugars and organic acids in strawberry fruits. ISHS Acta Hortic.

[CR34] Li H, Handsaker B, Wysoker A, Fennell T, Ruan J, Homer N, Marth G, Abecasis G, Durbin R (2009). The sequence alignment/map format and SAMtools. Bioinformatics.

[CR35] Maccaferri M, Ratti C, Rubies-Autonell C, Vallega V, Demontis A, Stefanelli S, Tuberosa R, Sanguineti MC (2011). Resistance to Soil-borne cereal mosaic virus in durum wheat is controlled by a major QTL on chromosome arm 2BS and minor loci. Theor Appl Genet.

[CR36] Mao D, Liu T, Xu C, Li X, Xing Y (2011). Epistasis and complementary gene action adequately account for the genetic bases of transgressive segregation of kilo-grain weight in rice. Euphytica.

[CR37] Ming R, Liu SC, Moore PH, Irvine JE, Paterson AH (2001). QTL analysis in a complex autopolyploid: genetic control of sugar content in sugarcane. Genome Res.

[CR38] Paran I, van der Knaap E (2007). Genetic and molecular regulation of fruit and plant domestication traits in tomato and pepper. J Exp Bot.

[CR39] Pattison RJ, Csukasi F, Catalá C (2014). Mechanisms regulating auxin action during fruit development. Physiol Plant.

[CR40] Peleg Z, Fahima T, Krugman T, Abbo S, Yakir D, Korol AB, Saranga Y (2009). Genomic dissection of drought resistance in durum wheat × wild emmer wheat recombinant inbreed line population. Plant Cell Environ.

[CR41] Pott DM, Vallarino JG, Osorio S, Amaya I (2018). Fruit ripening and QTL for fruit quality in the octoploid strawberry. In: The genomes of rosaceous berries and their wild relatives.

[CR42] Potter D, Luby JJ, Harrison RE (2000). Phylogenetic relationships among sepcies of fragaria (Rosaceae) inferred from non-coding nuclear and chloroplast DNA sequences. Syst Bot.

[CR43] Quilot B, Wu BH, Kervella J, Génard M, Foulongne M, Moreau K (2004). QTL analysis of quality traits in an advanced backcross between Prunus persica cultivars and the wild relative species P. davidiana. Theor Appl Genet.

[CR44] Ring L, Yeh S-Y, Hücherig S, Hoffmann T, Blanco-Portales R, Fouche M, Villatoro C, Denoyes B, Monfort A, Caballero JL (2013). Metabolic interaction between anthocyanin and lignin biosynthesis is associated with peroxidase FaPRX27 in strawberry fruit. Plant Physiol.

[CR45] Rousseau-Gueutin M, Lerceteau-Köhler E, Barrot L, Sargent DJ, Monfort A, Simpson D, Arús P, Guérin G, Denoyes-Rothan B (2008). Comparative genetic mapping between octoploid and diploid fragaria species reveals a high level of colinearity between their genomes and the essentially disomic behavior of the cultivated octoploid strawberry. Genetics.

[CR46] Rousseau-Gueutin M, Gaston A, Aïnouche A, Aïnouche ML, Olbricht K, Staudt G, Richard L, Denoyes-Rothan B (2009). Tracking the evolutionary history of polyploidy in Fragaria L. (strawberry): new insights from phylogenetic analyses of low-copy nuclear genes. Mol Phylogenet Evol.

[CR47] Saliba-Colombani V, Causse M, Langlois D, Philouze J, Buret M (2001). Genetic analysis of organoleptic quality in fresh market tomato. 1. Mapping QTLs for physical and chemical traits. TAG Theor Appl Genet.

[CR48] Sánchez-Sevilla JF, Horvath A, Botella MA, Gaston A, Folta K, Kilian A, Denoyes B, Amaya I (2015). Diversity arrays technology (DArT) marker platforms for diversity analysis and linkage mapping in a complex crop, the octoploid cultivated strawberry (Fragaria × ananassa). PLoS ONE.

[CR49] Sargent DJ, Fernandéz-Fernandéz F, Ruiz-Roja JJ, Sutherland BG, Passey A, Whitehouse AB, Simpson DW (2009). A genetic linkage map of the cultivated strawberry (fragaria × ananassa) and its comparison to the diploid fragaria reference map. Mol Breed.

[CR50] Sargent DJ, Passey T, Šurbanovski N, Lopez Girona E, Kuchta P, Davik J, Harrison R, Passey A, Whitehouse AB, Simpson DWA (2012). microsatellite linkage map for the cultivated strawberry (Fragaria x ananassa) suggests extensive regions of homozygosity in the genome that may have resulted from breeding and selection. Theor Appl Genet.

[CR51] Sargent DJ, Yang Y, Surbanovski N, Bianco L, Buti M, Velasco R, Giongo L, Davis TM (2016). HaploSNP affinities and linkage map positions illuminate subgenome composition in the octoploid, cultivated strawberry (Fragariaxananassa). Plant Sci.

[CR52] Schmieder R, Edwards R (2011). Quality control and preprocessing of metagenomic datasets. Bioinformatics.

[CR53] Shanmugam A, Hossain MR, Natarajan S, Jung H-J, Song J-Y, Kim H-T, Nou I-S (2017). Sugar content analysis and expression profiling of sugar related genes in contrasting Strawberry (Fragaria × ananassa) cultivars. J Plant Biotechnol.

[CR54] Shaw DV (1997). Trait mean depression for second-generation inbred strawberry populations with and without parent selection. Theor Appl Genet.

[CR55] Shirasawa K, Tanaka M, Takahata Y, Ma D, Cao Q, Liu Q, Zhai H, Kwak SS, Cheol Jeong J, Yoon UH (2017). A high-density SNP genetic map consisting of a complete set of homologous groups in autohexaploid sweetpotato (Ipomoea batatas). Sci Rep.

[CR56] Shulaev V, Sargent DJ, Crowhurst RN, Mockler TC, Folkerts O, Delcher AL, Jaiswal P, Mockaitis K, Liston A, Mane SP (2011). The genome of woodland strawberry (Fragaria vesca). Nat Genet.

[CR57] Spigler RB, Lewers KS, Johnson AL, Ashman T-L (2010). Comparative mapping reveals autosomal origin of sex chromosome in octoploid fragaria virginiana. J Hered.

[CR58] Stepanova AN, Yun J, Robles LM, Novak O, He W, Guo H, Ljung K, Alonso JM (2011). The *Arabidopsis* YUCCA1 flavin monooxygenase functions in the indole-3-pyruvic acid branch of auxin biosynthesis. Plant Cell.

[CR59] Sugimoto T, Tamaki K, Matsumoto J, Yamamoto Y, Shiwaku K, Watanabe K (2005). Detection of RAPD markers linked to the everbearing gene in Japanese cultivated strawberry. Plant Breed.

[CR60] Tamba CL, Ni Y-L, Zhang Y-M (2017). Iterative sure independence screening EM-Bayesian LASSO algorithm for multi-locus genome-wide association studies. PLoS Comput Biol.

[CR61] Tennessen JA, Govindarajulu R, Ashman TL, Liston A (2014). Evolutionary origins and dynamics of octoploid strawberry subgenomes revealed by dense targeted capture linkage maps. Genome Biol Evol.

[CR62] Vallarino JG, Pott DM, Cruz-Rus E, Miranda L, Medina-Minguez JJ, Valpuesta V, Fernie AR, Sánchez-Sevilla JF, Osorio S, Amaya I (2019). Identification of quantitative trait loci and candidate genes for primary metabolite content in strawberry fruit. Hortic Res.

[CR63] van Dijk T, Pagliarani G, Pikunova A, Noordijk Y, Yilmaz-Temel H, Meulenbroek B, Visser RGF, Van de Weg E (2014). Genomic rearrangements and signatures of breeding in the allo-octoploid strawberry as revealed through an allele dose based SSR linkage map. BMC Plant Biol.

[CR64] Van Ooijen JW (2006) JoinMap^®^ 4, Software for the calculation of genetic linkage maps in experimental populations. Kyazma BV, Wageningen, pp 33.

[CR65] Verma S, Zurn JD, Salinas N, Mathey MM, Denoyes B, Hancock JF, Finn CE, Bassil NV, Whitaker VM (2017). Clarifying sub-genomic positions of QTLs for flowering habit and fruit quality in US strawberry (Fragariaananassa) breeding populations using pedigree-based QTL analysis. Hortic Res.

[CR66] Vining KJ, Salinas N, Tennessen JA, Zurn JD, Sargent DJ, Hancock J, Bassil NV (2017). Genotyping-by-sequencing enables linkage mapping in three octoploid cultivated strawberry families. PeerJ.

[CR67] Wang S-B, Feng J-Y, Ren W-L, Huang B, Zhou L, Wen Y-J, Zhang J, Dunwell JM, Xu S, Zhang Y-M (2016). Improving power and accuracy of genome-wide association studies via a multi-locus mixed linear model methodology. Sci Rep.

[CR68] Weebadde CK, Wang D, Finn CE, Lewers KS, Luby JJ, Bushakra J, Sjulin TM, Hancock JF (2008). Using a linkage mapping approach to identify QTL for day-neutrality in the octoploid strawberry. Plant Breed.

[CR69] Wen Y-J, Zhang H, Ni Y-L, Huang B, Zhang J, Feng J-Y, Wang S-B, Dunwell JM, Zhang Y-M, Wu R (2018). Methodological implementation of mixed linear models in multi-locus genome-wide association studies. Brief Bioinform.

[CR70] Zhang J, Feng JY, Ni YL, Wen YJ, Niu Y, Tamba CL, Yue C, Song Q, Zhang YM (2017). pLARmEB: integration of least angle regression with empirical Bayes for multilocus genome-wide association studies. Heredity (Edinb).

[CR71] Zorrilla-Fontanesi Y, Cabeza A, Domínguez P, Medina JJ, Valpuesta V, Denoyes-Rothan B, Sánchez-Sevilla JF, Amaya I (2011). Quantitative trait loci and underlying candidate genes controlling agronomical and fruit quality traits in octoploid strawberry (Fragaria × ananassa). Theor Appl Genet.

