# Supplementary material for: ddRAD-seq derived genome-wide SNPs, high density linkage map and QTLs for fruit quality traits in strawberry (Fragaria x ananassa)
Source: 3 Biotech. 2020 Jul 27;10(8):353. doi: 10.1007/s13205-020-02291-5 (PMC7385052; doi:10.1007/s13205-020-02291-5)
Supplement: Supplementary file 1 — Supplementary Materials: The following are available online at www.mdpi.com/xxx/s1 (DOCX 463 kb) [file 13205_2020_2291_MOESM1_ESM.docx]

**Supplementary information**

**ddRAD-seq derived genome-wide SNPs, high density linkage map and QTLs for fruit quality traits in strawberry (*Fragaria x ananassa*)**

**Sathishkumar Natarajan^‡^, Mohammad Rashed Hossain^‡^,** **Hoy-Taek Kim*, Denison Michael Immanuel Jesse, Mostari Jahan Ferdous, Hee-Jeong Jung, Jong-In Park, Ill-Sup Nou***

Department of Horticulture, Suncheon National University, 255 Jungang-ro, Suncheon, Jeonnam 57922, Republic of Korea

* Correspondence: nis@sunchon.ac.kr (I.-S.N.), htkim@sunchon.ac.kr (H.-T.K.), Tel.: +82617503242 (H.-T.K); Tel.: +82-617-503-249 (I.-S.N.); Fax: +82-617-505-389 (H.-T.K. & I.-S.N.)

^‡^ These authors contributed equally to this work.

**
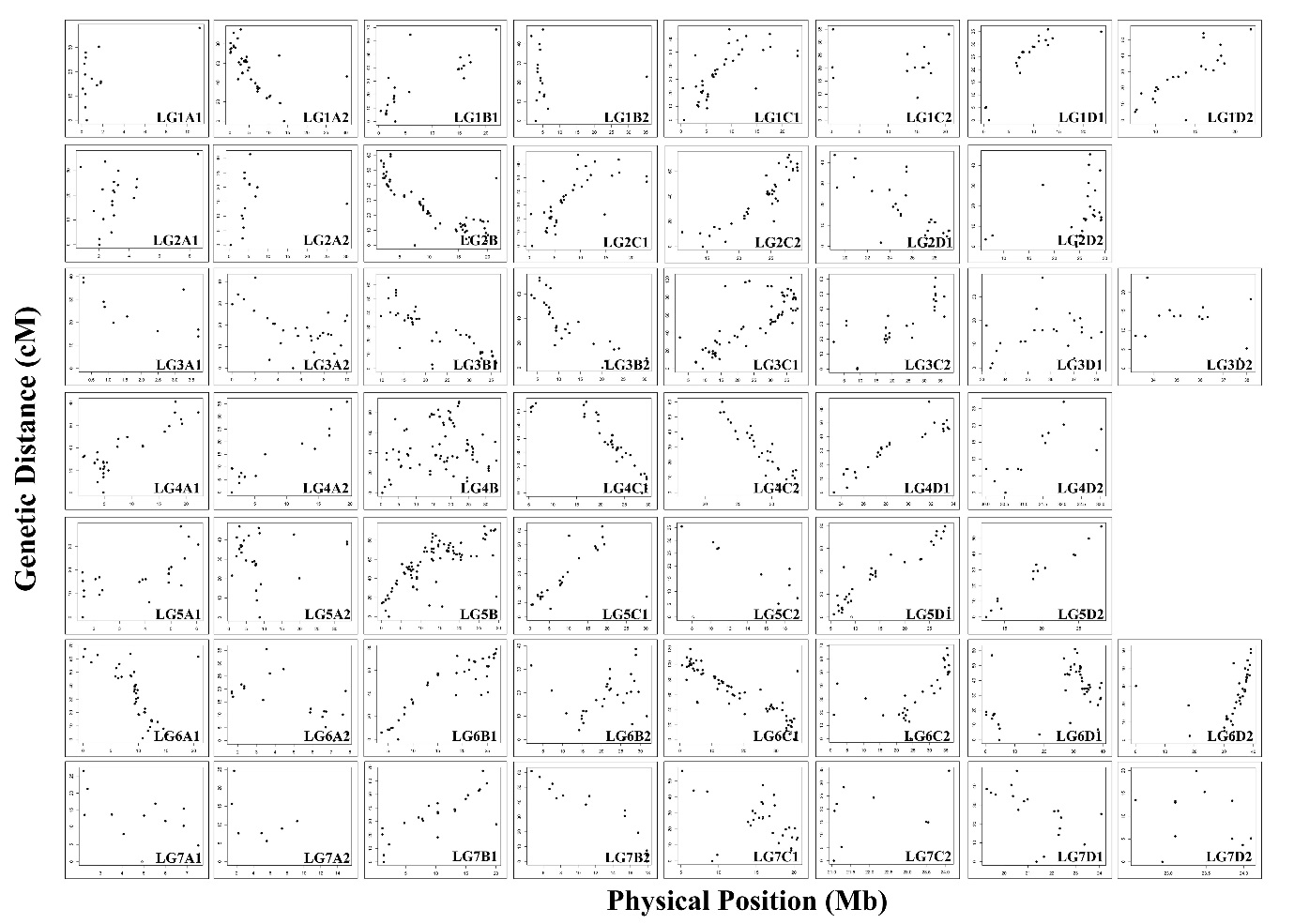
**

## Figure S1. Marey maps showing the alignment of the genetic positions of the SNPs on the constructed genetic linkage map with regards to the physical positions (Mb) of the SNP-marker associate sequence tags on the *F. vesca* genome (v4.0.a1).

## Figure S2: Allelic effect of the Fruit soluble solid content (SSC) related High resolution melting marker MF154 derived SNPs on Fruit weight for each genotype of F_1_ segregating population. The colored arrows indicate the mean fruit weight for the respective alleles carried by each genotype.

## Table S1. Statistical summary of barcodes, raw reads, mapped reads and reference genome alignment rate of each strawberry genotypes sequenced by ddRAD-sequencing technique.

| **SL** | **Samples** | **Barcodes** | **Raw reads** | **Mapped to reference genome** | | | |
| --- | --- | --- | --- | --- | --- | --- | --- |
|  |  |  |  | **Forward reads** | **Reverse reads** | **Total** | **Alignment rate** |
| 1 | Maehyang (M)-01 | TAAGGCGA | 1296564 | 931300 | 123449 | 1054749 | 81.35% |
| 2 | Maehyang (M)-02 | CGTACTAG | 1045339 | 746622 | 102425 | 849047 | 81.22% |
| 3 | Maehyang (M)-03 | AGGCAGAA | 1083038 | 785758 | 102730 | 888488 | 82.04% |
| 4 | Festival (A)-01 | TCCTGAGC | 1133327 | 814548 | 109282 | 923830 | 81.51% |
| 5 | Festival (A)-02 | GGACTCCT | 1177768 | 805260 | 121686 | 926946 | 78.70% |
| 6 | Festival (A)-03 | TAGGCATG | 1061738 | 728402 | 106919 | 835321 | 78.67% |
| 7 | M x F-01 | CACGCGAG | 1220140 | 881216 | 121295 | 1002511 | 82.16% |
| 8 | M x F-02 | ACGTATCA | 1437623 | 1032844 | 137421 | 1170265 | 81.40% |
| 9 | M x F-03 | CTACTATG | 1509741 | 1062194 | 153999 | 1216193 | 80.56% |
| 10 | M x F-04 | GCTACGCT | 1383596 | 1007534 | 131227 | 1138761 | 82.30% |
| 11 | M x F-05 | CGAGGCTG | 1202773 | 842774 | 120060 | 962834 | 80.05% |
| 12 | M x F-06 | AAGAGGCA | 1630530 | 1192926 | 148479 | 1341405 | 82.27% |
| 13 | M x F-07 | GTAGAGGA | 1278733 | 909748 | 126810 | 1036558 | 81.06% |
| 14 | M x F-08 | CGCGATAT | 1443281 | 1037400 | 145992 | 1183392 | 81.99% |
| 15 | M x F-09 | CACGCGAG | 1201986 | 865258 | 120963 | 986221 | 82.05% |
| 16 | M x F-10 | ACGTATCA | 1580595 | 1114486 | 156055 | 1270541 | 80.38% |
| 17 | M x F-11 | CTACTATG | 1591200 | 1146426 | 157811 | 1304237 | 81.97% |
| 18 | M x F-12 | GCTACGCT | 1481231 | 1042798 | 150091 | 1192889 | 80.53% |
| 19 | M x F-13 | CGAGGCTG | 1348529 | 957822 | 131182 | 1089004 | 80.75% |
| 20 | M x F-14 | AAGAGGCA | 1222582 | 877948 | 118983 | 996931 | 81.54% |
| 21 | M x F-15 | GTAGAGGA | 1371300 | 929668 | 152461 | 1082129 | 78.91% |
| 22 | M x F-16 | CGCGATAT | 1427461 | 980782 | 148572 | 1129354 | 79.12% |
| 23 | M x F-17 | CACGCGAG | 1200889 | 824892 | 126086 | 950978 | 79.19% |
| 24 | M x F-18 | ACGTATCA | 1407110 | 977492 | 141190 | 1118682 | 79.50% |
| 25 | M x F-19 | CTACTATG | 1462477 | 998834 | 159529 | 1158363 | 79.21% |
| 26 | M x F-20 | GCTACGCT | 1323206 | 878648 | 149215 | 1027863 | 77.68% |
| 27 | M x F-21 | CGAGGCTG | 1310864 | 884446 | 139609 | 1024055 | 78.12% |
| 28 | M x F-22 | AAGAGGCA | 1305965 | 887024 | 137885 | 1024909 | 78.48% |
| 29 | M x F-23 | GTAGAGGA | 1705499 | 1233112 | 162414 | 1395526 | 81.83% |
| 30 | M x F-24 | CGCGATAT | 1363052 | 970262 | 133257 | 1103519 | 80.96% |
| 31 | M x F-25 | CACGCGAG | 1198710 | 850946 | 120696 | 971642 | 81.06% |
| 32 | M x F-26 | ACGTATCA | 1490140 | 1025788 | 155534 | 1181322 | 79.28% |
| 33 | M x F-27 | CTACTATG | 1376745 | 984612 | 138255 | 1122867 | 81.56% |
| 34 | M x F-28 | GCTACGCT | 1347665 | 952978 | 145906 | 1098884 | 81.54% |
| 35 | M x F-29 | CGAGGCTG | 1306227 | 941434 | 126486 | 1067920 | 81.76% |
| 36 | M x F-30 | AAGAGGCA | 1426610 | 1021874 | 138324 | 1160198 | 81.33% |
| 37 | M x F-31 | GTAGAGGA | 1433752 | 970920 | 156390 | 1127310 | 78.63% |
| 38 | M x F-32 | CGCGATAT | 1424381 | 1030348 | 141407 | 1171755 | 82.26% |
| 39 | M x F-33 | CACGCGAG | 1303810 | 943636 | 130233 | 1073869 | 82.36% |
| 40 | M x F-34 | ACGTATCA | 1644281 | 1200796 | 158020 | 1358816 | 82.64% |
| 41 | M x F-35 | CTACTATG | 1603710 | 1142024 | 164109 | 1306133 | 81.44% |
| 42 | M x F-36 | GCTACGCT | 1264339 | 915524 | 121363 | 1036887 | 82.01% |
| 43 | M x F-37 | CGAGGCTG | 1064057 | 767918 | 105775 | 873693 | 82.11% |
| 44 | M x F-38 | AAGAGGCA | 1296193 | 940644 | 125018 | 1065662 | 82.21% |
| 45 | M x F-39 | GTAGAGGA | 1442452 | 1030100 | 143970 | 1174070 | 81.39% |
| 46 | M x F-40 | CGCGATAT | 1311187 | 927472 | 133498 | 1060970 | 80.92% |
| 47 | M x F-41 | CACGCGAG | 1318603 | 934018 | 135540 | 1069558 | 81.11% |
| 48 | M x F-42 | ACGTATCA | 1115182 | 783884 | 126161 | 910045 | 81.61% |
| 49 | M x F-43 | CTACTATG | 1339383 | 959790 | 134478 | 1094268 | 81.70% |

## Table S2: Provided in separate spread sheet (accessible from journal website online).

## Table S3: Provided in separate spread sheet (accessible from journal website online).

## Table S4: Provided in separate spread sheet (accessible from journal website online).

## Table S5: Phenotypic correlations among the fruit quality traits of strawberry as measured by Pearson's correlation coefficient analysis in the F1 progeny.

| **Traits** | **Fruit Length** | **Fruit Diameter** | **Fruit Weight** |
| --- | --- | --- | --- |
| **Fruit Diameter** | 0.236 (0.091) |  |  |
| **Fruit Weight** | 0.481 (0.025) | 0.512 (0.003) |  |
| **Fruit SSC** | -0.582 (0.008) | -0.241 (0.068) | -0.613 (0.003) |

Values without parenthesis indicate correlation coefficient, while values in parenthesis indicate corresponding P-values. SSC. Fruit soluble solid content.

## Table S6: Provided in separate spread sheet (accessible from journal website online).

**Table S7**: QTL flanking markers along with associated gene IDs and putative functions. The *Fragaria* x *ananassa* genome IDs corresponding to the flanking markers of the QTLs were first blasted against *Fragaria vesca* annotated genome version v2.0.a1 and the corresponding putative functions are listed.

| **QTL** | **Marker** | ***Fragaria × ananassa ID_SNP position*** | ***Fragaria vesca* *g*ene ID & annotation** | **Positional variant** |
| --- | --- | --- | --- | --- |
| ***QTLs for soluble sugar content*** | | |  |  |
| ***qSSC-5A2.1*** | MF1180 | FAN_icon20112375.1_294 | >gene32174-v1.0-hybrid Protein EXECUTER 1, chloroplastic, Precursor | missense_variant |
| ***qSSC-5A2.1*** | MF518 | FAN_iscf00158336.1_736 | >gene26006-v1.0-hybrid Hybrid signal transduction histidine kinase D | 3_prime_UTR_variant |
| ***qSSC-2B*** | MF994 | FAN_iscf00135334.1_10024 | >gene01635-v1.0-hybrid Transcriptional activator DEMETER (probable) | synonymous_variant |
| ***qSSC-2B*** | MF1321 | FAN_iscf00286716.1_2795 | >gene31913-v1.0-hybrid Pathogenesis-related protein 1A (PR-1A) | upstream_gene_variant |
| ***qSSC-2B*** | MF472 | FAN_iscf00049825.1_1571 | >gene26249-v1.0-hybrid Limonoid UDP-glucosyltransferase | upstream_gene_variant |
| ***qSSC-5A2.2*** | MF811 | FAN_iscf00290095.1_17 | >gene09526-v1.0-hybrid hypothetical protein | missense_variant |
| ***qSSC-5A2.2*** | MF1169 | FAN_icon19417585.1_76 | >gene32383-v1.0-hybrid Multidrug and toxin extrusion protein 1 | intergenic_region |
| ***qSSC-1A2*** | MF3293 | FAN_iscf00062613.1_5856 | >gene14770-v1.0-hybrid AP-3 complex subunit sigma-2 (similar to) | upstream_gene_variant |
| ***qSSC-1A2*** | MF3696 | FAN_iscf00100711.1_5212 | >gene14732-v1.0-hybrid Soluble inorganic pyrophosphatase (PPase) | 5_prime_UTR_variant |
| ***QTLs for fruit length*** | |  |  |  |
| ***qFL-6C1*** | MF3023 | FAN_iscf00104879.1_1513 | >gene07414-v1.0-hybrid Transcription initiation factor TFIID subunit 12 | missense_variant |
| ***qFL-6C1*** | MF2522 | FAN_iscf00160810.1_3089 | ">gene15527-v1.0-hybrid Glucose-6-phosphate 1-dehydrogenase, | missense_variant |
| ***qFL-7C1*** | MF2965 | FAN_iscf00037535.1_172 | >gene23263-v1.0-hybrid Extended synaptotagmin-2-B (E-Syt2-B) | intergenic_region |
| ***qFL-7C1*** | MF2544 | FAN_iscf00229100.1_103 | >gene03090-v1.0-hybrid Dynamin-related protein 1E (similar to) | missense_variant |
| ***qFL-3B1*** | MF2104 | FAN_iscf00387732.1_1589 | >gene27177-v1.0-hybrid Serine/arginine repetitive matrix protein 1 | intergenic_region |
| ***qFL-3B1*** | MF408 | FAN_icon19954809.1_297 | >gene22043-v1.0-hybrid Poly [ADP-ribose] polymerase 3 (PARP-3) | intron_variant |
| ***qFL-4A2*** | MF3389 | FAN_iscf00160711.1_163 | N/A | intergenic_region |
| ***qFL-4A2*** | MF3754 | FAN_icon19840611.1_216 | N/A | intergenic_region |
| ***qFL-5C2*** | MF3498 | FAN_iscf00331071.1_286 | >gene29218-v1.0-hybrid Filaggrin (probable) | missense_variant |
| ***qFL-5C2*** | MF2834 | FAN_iscf00088923.1_5346 | >gene35225-v1.0-hybrid Copper-transporting ATPase (probable) | missense_variant |
| ***qFL-7A2*** | MF2721 | FAN_iscf00008836.1_480 | >gene04808-v1.0-hybrid DnaJ protein homolog 2 (similar to) | stop_gained |
| ***qFL-7A2*** | MF2920 | FAN_icon19998173.1_277 | >gene12113-v1.0-hybrid Ubiquitin carboxyl-terminal hydrolase 16 | intergenic_region |
| ***QTLs for fruit diameter*** | |  |  |  |
| ***qFD-4C2*** | MF2741 | FAN_iscf00051427.1_1734 | >gene25324-v1.0-hybrid Putative AC transposase (probable) | intergenic_region |
| ***qFD-4C2*** | MF3597 | FAN_iscf00078828.1_646 | >gene00638-v1.0-hybrid Probable ubiquitin thiolesterase DG1039 | synonymous_variant |
| ***qFD-2B*** | MF1601 | FAN_iscf00275054.1_208 | >gene21830-v1.0-hybrid Transcription factor TFIIIB component B'' | missense_variant |
| ***qFD-2B*** | MF994 | FAN_iscf00135334.1_10024 | >gene01635-v1.0-hybrid Transcriptional activator DEMETER (probable) | synonymous_variant |
| ***qFD-1B2*** | MF1848 | FAN_iscf00019693.1_9132 | >gene11984-v1.0-hybrid Nucleocapsid protein p15-gag (similar to) | downstream_gene_variant |
| ***qFD-1B2*** | MF2390 | FAN_iscf00346482.1_545 | >gene12647-v1.0-hybrid Translation machinery-associated protein 22 | splice_region_variant&  intron_variant |
| ***qFD-4B*** | MF2448 | FAN_icon20620372.1_603 | N/A | intergenic_region |
| ***qFD-4B*** | MF1306 | FAN_iscf00244831.1_4826 | >gene07162-v1.0-hybrid Sulfite oxidase (At-SO) (putative) | downstream_gene_variant |
| ***QTLs for fruit weight*** | |  |  |  |
| ***qFW-1B2.1*** | MF34 | FAN_iscf00071525.1_8985 | >gene12732-v1.0-hybrid 40S ribosomal protein S3-3 (putative) | splice_region_variant&  intron_variant |
| ***qFW-1B2.1*** | MF1261 | FAN_iscf00128046.1_7877 | >gene12714-v1.0-hybrid Protein Brevis radix-like 3 (AtBRXL3) | downstream_gene_variant |
| ***qFW-1B2.1*** | MF1523 | FAN_iscf00150808.1_4380 | ">gene12920-v1.0-hybrid Beta-galactosidase 15 (Lactase 15) | upstream_gene_variant |
| ***qFW-1B2.2*** | MF1848 | FAN_iscf00019693.1_9132 | >gene11984-v1.0-hybrid Nucleocapsid protein p15-gag (similar to) | downstream_gene_variant |
| ***qFW-1B2.2*** | MF1619 | FAN_iscf00290643.1_840 | N/A | intergenic_region |
| ***qFW-4B*** | MF818 | FAN_iscf00345769.1_930 | >gene04013-v1.0-hybrid DNA-binding protein HEXBP (probable) | 3_prime_UTR_variant |
| ***qFW-4B*** | MF1306 | FAN_iscf00244831.1_4826 | >gene07162-v1.0-hybrid Sulfite oxidase (At-SO) (putative) | downstream_gene_variant |
| ***qFW-6A2*** | MF332 | FAN_iscf00266073.1_572 | ">gene18377-v1.0-hybrid Putative receptor protein kinase ZmPK1, | missense_variant |
| ***qFW-6A2*** | MF2398 | FAN_iscf00355876.1_479 | >gene13460-v1.0-hybrid Stress protein DDR48 (DDRP 48) (probable) | 3_prime_UTR_variant |

**Table S8:** Sugar biosynthesis related genes and the transcription regulator activity genes manually extracted from the total genes that lie within the region of four sugar related QTLs.

| **S/N** | **Gene ID** | **Location** | **Annotation** |
| --- | --- | --- | --- |
| ***Sugar related genes*** | |  |  |
| 1 | FvH4_5g13450.1 | Fvb5_v4.0.a1:7600019..7603547 | galacturonosyltransferase 11 |
| 2 | FvH4_5g13650.1 | Fvb5_v4.0.a1:7719651..7725499 | Galactose oxidase/kelch repeat superfamily protein |
| 3 | FvH4_5g10320.1 | Fvb5_v4.0.a1:5897388..5899700 | Nucleotide-diphospho-sugar transferases superfamily protein |
| 4 | FvH4_5g10510.1 | Fvb5_v4.0.a1:5980120..5984212 | Galactosyltransferase family protein |
| 5 | FvH4_5g14300.1 | Fvb5_v4.0.a1:8088754..8090787 | Rhamnogalacturonate lyase family protein |
| 6 | FvH4_5g08750.1 | Fvb5_v4.0.a1:5017636..5020804 | SWITCH/sucrose nonfermenting 3A |
| 7 | FvH4_5g14230.1 | Fvb5_v4.0.a1:8024429..8026587 | UDP-glucose 6-dehydrogenase family protein |
| 8 | FvH4_5g07300.1 | Fvb5_v4.0.a1:4273691..4281218 | Galactose oxidase/kelch repeat superfamily protein |
| 9 | FvH4_5g15890.1 | Fvb5_v4.0.a1:8988231..8992181 | Galactosyltransferase family protein |
| 10 | FvH4_5g10320.1 | Fvb5_v4.0.a1:5897388..5899700 | Nucleotide-diphospho-sugar transferases superfamily protein |
| 11 | FvH4_5g08750.1 | Fvb5_v4.0.a1:5017636..5020804 | SWITCH/sucrose nonfermenting 3A |
| 12 | FvH4_5g05210.1 | Fvb5_v4.0.a1:3032458..3036151 | SUGAR-INSENSITIVE 3 |
| 13 | FvH4_5g04740.1 | Fvb5_v4.0.a1:2778196..2778375 | UDP-glucose 4-epimerase GEPI48-like |
| 14 | FvH4_5g05430.1 | Fvb5_v4.0.a1:3189242..3192898 | Glucose-6-phosphate/phosphate translocator-related |
| 15 | FvH4_5g07360.1 | Fvb5_v4.0.a1:4303994..4306138 | Plant neutral invertase family protein |
| 16 | FvH4_5g07480.1 | Fvb5_v4.0.a1:4368462..4370602 | Plant neutral invertase family protein |
| 17 | FvH4_5g14710.1 | Fvb5_v4.0.a1:8349749..8351377 | Plant invertase/pectin methylesterase inhibitor superfamily |
| 18 | FvH4_5g07360.1 | Fvb5_v4.0.a1:4303994..4306138 | Plant neutral invertase family protein |
| 19 | FvH4_5g07480.1 | Fvb5_v4.0.a1:4368462..4370602 | Plant neutral invertase family protein |
| 20 | FvH4_5g14710.1 | Fvb5_v4.0.a1:8349749..8351377 | Plant invertase/pectin methylesterase inhibitor superfamily |
| 21 | FvH4_5g14710.1 | Fvb5_v4.0.a1:8349749..8351377 | Plant invertase/pectin methylesterase inhibitor superfamily |
| ***Transcription regulator activity genes*** | | |  |
| 1 | FvH4_1g13230.1 | Fvb1_v4.0.a1:7269666..7271203 | GATA type zinc finger transcription factor family protein |
| 2 | FvH4_1g13740.1 | Fvb1_v4.0.a1:7579905..7581563 | GRAS family transcription factor |
| 3 | FvH4_1g13400.1 | Fvb1_v4.0.a1:7344745..7347252 | GRAS family transcription factor |
| 4 | FvH4_1g13240.1 | Fvb1_v4.0.a1:7269725..7270012 | PREDICTED: GATA transcription factor 18-like [Fragaria vesca subsp. vesca] |
| 5 | FvH4_5g15050.1 | Fvb5_v4.0.a1:8520018..8522358 | transcription regulators;zinc ion binding |
| 6 | FvH4_5g12740.1 | Fvb5_v4.0.a1:7194196..7197438 | Transcription factor TFIIE, alpha subunit |
| 7 | FvH4_5g12750.1 | Fvb5_v4.0.a1:7197802..7202667 | Transcription factor TFIIE, alpha subunit |
| 8 | FvH4_5g13520.1 | Fvb5_v4.0.a1:7643263..7647739 | Squamosa promoter-binding protein-like (SBP domain) transcription factor family protein |
| 9 | FvH4_5g15150.1 | Fvb5_v4.0.a1:8560313..8561584 | TCP family transcription factor |
| 10 | FvH4_5g13630.1 | Fvb5_v4.0.a1:7712747..7714084 | Mitochondrial transcription termination factor family protein |
| 11 | FvH4_5g10690.1 | Fvb5_v4.0.a1:6071847..6073778 | NAC (No Apical Meristem) domain transcriptional regulator superfamily protein |
| 12 | FvH4_5g10410.1 | Fvb5_v4.0.a1:5929275..5930672 | AP2/B3-like transcriptional factor family protein |
| 13 | FvH4_5g14290.1 | Fvb5_v4.0.a1:8077517..8081561 | AP2/B3-like transcriptional factor family protein |
| 14 | FvH4_5g12710.1 | Fvb5_v4.0.a1:7171448..7173747 | TCP family transcription factor |
| 15 | FvH4_5g15310.1 | Fvb5_v4.0.a1:8679648..8680648 | global transcription factor group E8 |
| 16 | FvH4_5g08520.1 | Fvb5_v4.0.a1:4916144..4916335 | K-box region and MADS-box transcription factor family protein |
| 17 | FvH4_5g10350.1 | Fvb5_v4.0.a1:5903211..5906949 | NAC transcription factor-like 9 |
| 18 | FvH4_5g07880.1 | Fvb5_v4.0.a1:4582086..4583141 | Mitochondrial transcription termination factor family protein |
| 19 | FvH4_5g13510.1 | Fvb5_v4.0.a1:7627174..7634061 | K-box region and MADS-box transcription factor family protein |
| 20 | FvH4_5g08580.1 | Fvb5_v4.0.a1:4940285..4944053 | Squamosa promoter-binding protein-like (SBP domain) transcription factor family protein |
| 21 | FvH4_5g13710.1 | Fvb5_v4.0.a1:7753525..7754772 | TEOSINTE BRANCHED 1, cycloidea and PCF transcription factor 5 |
| 22 | FvH4_5g07280.1 | Fvb5_v4.0.a1:4258733..4265962 | AP2/B3-like transcriptional factor family protein |
| 23 | FvH4_5g15160.1 | Fvb5_v4.0.a1:8561300..8561653 | PREDICTED: transcription factor TCP14 [Fragaria vesca subsp. vesca] |
| 24 | FvH4_5g12740.1 | Fvb5_v4.0.a1:7194196..7197438 | Transcription factor TFIIE, alpha subunit |
| 25 | FvH4_5g12750.1 | Fvb5_v4.0.a1:7197802..7202667 | Transcription factor TFIIE, alpha subunit |
| 26 | FvH4_5g13520.1 | Fvb5_v4.0.a1:7643263..7647739 | Squamosa promoter-binding protein-like (SBP domain) transcription factor family protein |
| 27 | FvH4_5g13630.1 | Fvb5_v4.0.a1:7712747..7714084 | Mitochondrial transcription termination factor family protein |
| 28 | FvH4_5g05980.1 | Fvb5_v4.0.a1:3519192..3519701 | NAC (No Apical Meristem) domain transcriptional regulator superfamily protein |
| 29 | FvH4_5g10690.1 | Fvb5_v4.0.a1:6071847..6073778 | NAC (No Apical Meristem) domain transcriptional regulator superfamily protein |
| 30 | FvH4_5g10410.1 | Fvb5_v4.0.a1:5929275..5930672 | AP2/B3-like transcriptional factor family protein |
| 31 | FvH4_5g14290.1 | Fvb5_v4.0.a1:8077517..8081561 | AP2/B3-like transcriptional factor family protein |
| 32 | FvH4_5g12710.1 | Fvb5_v4.0.a1:7171448..7173747 | TCP family transcription factor |
| 33 | FvH4_5g06160.1 | Fvb5_v4.0.a1:3622433..3623152 | AP2/B3-like transcriptional factor family protein |
| 34 | FvH4_5g08520.1 | Fvb5_v4.0.a1:4916144..4916335 | K-box region and MADS-box transcription factor family protein |
| 35 | FvH4_5g10350.1 | Fvb5_v4.0.a1:5903211..5906949 | NAC transcription factor-like 9 |
| 36 | FvH4_5g07880.1 | Fvb5_v4.0.a1:4582086..4583141 | Mitochondrial transcription termination factor family protein |
| 37 | FvH4_5g13510.1 | Fvb5_v4.0.a1:7627174..7634061 | K-box region and MADS-box transcription factor family protein |
| 38 | FvH4_5g08580.1 | Fvb5_v4.0.a1:4940285..4944053 | Squamosa promoter-binding protein-like (SBP domain) transcription factor family protein |
| 39 | FvH4_5g13710.1 | Fvb5_v4.0.a1:7753525..7754772 | TEOSINTE BRANCHED 1, cycloidea and PCF transcription factor 5 |
| 40 | FvH4_5g07280.1 | Fvb5_v4.0.a1:4258733..4265962 | AP2/B3-like transcriptional factor family protein |
| 41 | FvH4_5g15050.1 | Fvb5_v4.0.a1:8520018..8522358 | transcription regulators;zinc ion binding |
| 42 | FvH4_5g13520.1 | Fvb5_v4.0.a1:7643263..7647739 | Squamosa promoter-binding protein-like (SBP domain) transcription factor family protein |
| 43 | FvH4_5g15150.1 | Fvb5_v4.0.a1:8560313..8561584 | TCP family transcription factor |
| 44 | FvH4_5g13630.1 | Fvb5_v4.0.a1:7712747..7714084 | Mitochondrial transcription termination factor family protein |
| 45 | FvH4_5g14290.1 | Fvb5_v4.0.a1:8077517..8081561 | AP2/B3-like transcriptional factor family protein |
| 46 | FvH4_5g15310.1 | Fvb5_v4.0.a1:8679648..8680648 | global transcription factor group E8 |
| 47 | FvH4_5g13510.1 | Fvb5_v4.0.a1:7627174..7634061 | K-box region and MADS-box transcription factor family protein |
| 48 | FvH4_5g13710.1 | Fvb5_v4.0.a1:7753525..7754772 | TEOSINTE BRANCHED 1, cycloidea and PCF transcription factor 5 |

## Table S9: List of high resolution melting (HRM) markers designed for characterizing high vs low sugar containing strawberry genotypes along with their corresponding genotyping accuracy in the segregating F_1_ population (F_1_) and in commercial cultivars (CC).

| **SL** | **Gene ID** | **Marker Name** | **Sequence (5’-3’)** | **SNP** | **SNP position** | **Description** | **Genotyping efficiency (%)** |
| --- | --- | --- | --- | --- | --- | --- | --- |
| 1 | FAN_iscf00158336 | MF518 | F: ATCAGGCTAGCTCTAAAGAC  R: ACAACAGTTTTGTGATCAGA  P: CAGCAAAATGAT**A**GAAGAAAAGTGC | [A/W] | 736 | Alpha/beta-Hydrolases superfamily protein | **In F_1_ :** 52.42%  **In CC:** 51.38% |
| 2 | FAN_iscf00286716 | MF1321 | F: TGCAGTAAAGAACCATGCTC  R: CCTCAAGGCCAAGGGTCAAG  P: TTTTAAATCACT**G**TGTCAAAGAGTC | [G/K] | 2795 | PREDICTED: uncharacterized protein | **In F_1_ :** 57.32%  **In CC:** 53.27% |
| 3 | FAN_iscf00100711 | MF3696 | F: TAAAAAGGTAGACGCCTCGT  R: CTTTCAAAGAAGTCGAGAGC  P: TTCTCTATTCTG**G**GAAACAAACGAA | [S/G] | 5212 | PREDICTED: soluble inorganic pyrophosphatase-like | **In F_1_ :** 49.37%  **In CC:** 43.21% |
| 4. | FAN_icon19417585 | MF1169 | F: ACCAATTGGGAACTTCAAGC  R: CCGGTTGCTGGGTTATACAT  P: GAAAGTTACACTA**T**TGTTGATGATGGT | [T/Y] | 76 | PREDICTED: MATE efflux family protein 8-like | **In F_1_ :** 53.81%  **In CC:** 54.33% |
| 5 | FAN_iscf00021287 (*FaGlu15*) | MF154* | F: AACCGACCTTGTGAAAGCTG  R: GGCGTTGATGAGGAAGACTA  P: GTCAACGATGAC**A**GTGCTGAAACCG | [G/A] | 1723 | UDP-glucose 4-epimerase GEPI48-like | **In F_1_ :** 81.39%  **In CC:** 86.95% |

The SNP allele that is used in the HRM probe is underlined. W = A/T; K = G/T; S = G/C; Y = C/T. *the most efficient marker.

## Table S10. Genotyping the parental, F1 and commercial strawberry lines by the developed high resolution melting (HRM) marker MF154. LS and HS indicate low (<8% brix) and high (≥8% brix) sugar containing genotypes, respectively.

| **SL** | **Genotype** | **SNP Allele** | **Sugar Content (% brix)** | **HRM Phenotype** |
| --- | --- | --- | --- | --- |
| ***Parental population*** | | | | |
| 1 | Maehyang | A | 10.13 | HS |
| 2 | Festival | G | 6.17 | LS |
| ***F1 lines*** | | | | |
| 1 | M x F-01 | A | 11.2 | HS |
| 2 | M x F-02 | A | 8.69 | LS |
| 3 | M x F-03 | G | 6.78 | HS |
| 4 | M x F-04 | A | 10.59 | HS |
| 5 | M x F-05 | A | 9.43 | LS |
| 6 | M x F-06 | G | 5.7 | LS |
| 7 | M x F-07 | A | 12.68 | HS |
| 8 | M x F-08 | G | 4.59 | LS |
| 9 | M x F-09 | G | 6.27 | LS |
| 10 | M x F-10 | G | 6.89 | LS |
| 11 | M x F-11 | G | 7.08 | LS |
| 12 | M x F-12 | A | 9.81 | HS |
| 13 | M x F-13 | A | 10.57 | HS |
| 14 | M x F-14 | A | 8.68 | LS |
| 15 | M x F-15 | G | 6.91 | LS |
| 16 | M x F-16 | A | 9.68 | HS |
| 17 | M x F-17 | A | 8.67 | HS |
| 18 | M x F-18 | G | 4.82 | LS |
| 19 | M x F-19 | A | 9.79 | HS |
| 20 | M x F-20 | A | 8.39 | HS |
| 21 | M x F-21 | G | 6.75 | LS |
| 22 | M x F-22 | A | 9.78 | HS |
| 23 | M x F-23 | G | 7.39 | HS |
| 24 | M x F-24 | A | 8.19 | HS |
| 25 | M x F-25 | G | 6.12 | LS |
| 26 | M x F-26 | A | 13.1 | HS |
| 27 | M x F-27 | G | 4.98 | LS |
| 28 | M x F-28 | A | 8.94 | HS |
| 29 | M x F-29 | A | 9.83 | HS |
| 30 | M x F-30 | G | 6.85 | LS |
| 31 | M x F-31 | A | 8.07 | HS |
| 32 | M x F-32 | G | 7.69 | HS |
| 33 | M x F-33 | A | 9.38 | HS |
| 34 | M x F-34 | A | 8.69 | LS |
| 35 | M x F-35 | G | 6.94 | LS |
| 36 | M x F-36 | A | 10.86 | HS |
| 37 | M x F-37 | G | 7.56 | HS |
| 38 | M x F-38 | A | 9.43 | HS |
| 39 | M x F-39 | A | 8.67 | HS |
| 40 | M x F-40 | G | 4.87 | LS |
| 41 | M x F-41 | G | 6.29 | LS |
| 42 | M x F-42 | G | 7.95 | LS |
| 43 | M x F-43 | G | 7.18 | LS |
| ***Commercial cultivars*** | | | | |
| 1 | Selva | | 4.33 | LS |
| 2 | Sunrise | | 4.37 | LS |
| 3 | Laihou | | 4.37 | LS |
| 4 | Zepier | | 4.5 | LS |
| 5 | Cardinal | | 4.67 | LS |
| 6 | Portola | | 4.8 | LS |
| 7 | Columbia | | 4.87 | LS |
| 8 | Catskill | | 6.17 | HS |
| 9 | Gwanha | | 6.98 | LS |
| 10 | Sweet Charlie | | 7.03 | HS |
| 11 | Albion | | 7.59 | LS |
| 12 | Soelhyang | | 9.08 | LS |
| 13 | Tamyang | | 10.51 | HS |
| 14 | Juckyang | | 10.58 | LS |
| 15 | Alps | | 10.77 | HS |
| 16 | Toyotama | | 10.77 | LS |
| 17 | Summerberry | | 11.53 | HS |
| 18 | SBNK | | 11.89 | HS |
| 19 | Akihime | | 12.13 | HS |
| 20 | Kingsberry | | 12.27 | HS |
| 21 | Shasta | | 12.3 | HS |
| 22 | Arihyang | | 12.5 | HS |
| 23 | Doorihyang | | 12.9 | HS |

The commercial cultivars were grown and phenotyped for SSC in the green house facility of Damyang-gun Agricultural Technology Center, Damyang, South Korea. LH. Low sugar, HS. High sugar.

==()==
